# Chronic Low Dose Oral Exposure to Microcystin-LR Exacerbates Hepatic Injury in a Murine Model of Non-Alcoholic Fatty Liver Disease

**DOI:** 10.3390/toxins11090486

**Published:** 2019-08-23

**Authors:** Apurva Lad, Robin C. Su, Joshua D. Breidenbach, Paul M. Stemmer, Nicholas J. Carruthers, Nayeli K. Sanchez, Fatimah K. Khalaf, Shungang Zhang, Andrew L. Kleinhenz, Prabhatchandra Dube, Chrysan J. Mohammed, Judy A. Westrick, Erin L. Crawford, Dilrukshika Palagama, David Baliu-Rodriguez, Dragan Isailovic, Bruce Levison, Nikolai Modyanov, Amira F. Gohara, Deepak Malhotra, Steven T. Haller, David J. Kennedy

**Affiliations:** 1 Department of Medicine, University of Toledo, Toledo, OH 43614, USA; 2 Institute of Environmental Health Sciences, Eugene Applebaum College of Pharmacy and Health Sciences, Wayne State University, Detroit, MI 48201, USA; 3 Department of Chemistry, Wayne State University, Detroit, MI 48202, USA; 4 Department of Chemistry and Biochemistry, University of Toledo, Toledo, OH 43606, USA; 5 Department of Pediatrics, University of Toledo, Toledo, OH 43614, USA; 6 Department of Physiology and Pharmacology, University of Toledo, Toledo, OH 43614, USA; 7 Department of Pathology, University of Toledo, Toledo, OH 43614, USA

**Keywords:** Microcystin-LR, Non-alcoholic Fatty Liver Disease, No Observed Adverse Effect Level, Lepr^db^/J mice, hepatotoxicity, oxidative stress, TiO_2_ enriched phosphopeptides

## Abstract

Microcystins are potent hepatotoxins that have become a global health concern in recent years. Their actions in at-risk populations with pre-existing liver disease is unknown. We tested the hypothesis that the No Observed Adverse Effect Level (NOAEL) of Microcystin-LR (MC-LR) established in healthy mice would cause exacerbation of hepatic injury in a murine model (Lepr^db^/J) of Non-alcoholic Fatty Liver Disease (NAFLD). Ten-week-old male Lepr^db^/J mice were gavaged with 50 μg/kg, 100 μg/kg MC-LR or vehicle every 48 h for 4 weeks (*n* = 15–17 mice/group). Early mortality was observed in both the 50 μg/kg (1/17, 6%), and 100 μg/kg (3/17, 18%) MC-LR exposed mice. MC-LR exposure resulted in significant increases in circulating alkaline phosphatase levels, and histopathological markers of hepatic injury as well as significant upregulation of genes associated with hepatotoxicity, necrosis, nongenotoxic hepatocarcinogenicity and oxidative stress response. In addition, we observed exposure dependent changes in protein phosphorylation sites in pathways involved in inflammation, immune function, and response to oxidative stress. These results demonstrate that exposure to MC-LR at levels that are below the NOAEL established in healthy animals results in significant exacerbation of hepatic injury that is accompanied by genetic and phosphoproteomic dysregulation in key signaling pathways in the livers of NAFLD mice.

## 1. Introduction

Cyanobacteria are a natural component of the freshwater phytoplankton, but their overgrowth has increasingly been recognized as a potential health hazard due to the release of toxins by selected species of algae, such as *Microcystis aeruginosa*, *Raphidiopsis mediterranea, Cylindrospermopsin raciborskii*, *Anabaena circinalis, Planktothrix rubescens and Planktothrix agardhii* [1,2,3,4,5]. Their ability to adapt and flourish in a wide variety of climate conditions, including extremes, contributes to their successful occurrence in a variety of ecosystems. An extensive study done by Mantzouki et al. in 2018, showed that global warming has direct and indirect effects on the temperature and nutrient gradient in the freshwater systems, thus, affecting the distribution of the cyanotoxins [5]. Therefore, changing climatic conditions and increased eutrophication of freshwater ecosystems are two main factors that favor the growth of cyanobacterial blooms and allow them to flourish, increasing the duration and intensity on a global scale [2,6].

Toxin production is a common characteristic of many of the cyanobacterial species. Of the many toxins produced by cyanobacteria, microcystins (MCs) are among the most widely distributed, and microcystin-LR (MC-LR) is one of the most commonly produced [7]. MC-LR is a cyclic heptapeptide characterized structurally by leucine and arginine amino acids at positions 2 and 4 within a cyclo-(D-alanine-1-X2-D-MeAsp3-Y4-Adda 5-d-glutamate 6-Mdha7) structure [8]. It is usually taken up into the cells through organic anion transporting polypeptides (OATPs) [9]. MC-LR then exhibits its deleterious effects by inhibiting the activity of protein phosphatases 1 (PP1) and 2A (PP2A) and by increasing the production of reactive oxygen species (ROS) [10,11]. As a result, there is a hyperphosphorylation and pro-oxidative state induced by MC-LR, which leads to alterations in the cytoskeleton, OATP expression, mitogen-activated protein kinase (MAPK) activity, glycogen storage, and mitochondrial structure and function in addition to DNA damage, cellular disruption, endoplasmic reticulum dysfunction, inflammation and tumor growth [12,13,14,15,16,17,18,19,20].

MC-LR has become a global health concern due to its potent toxicity in humans and animals alike. In addition to its well-known hepatotoxic effects, MCs also cause reproductive, developmental and, immune toxicities [9,21,22,23,24,25,26,27,28]. Humans can be exposed to MC-LR most commonly through oral ingestion of contaminated water, or through recreational activities like swimming or boating in contaminated waters, as well as intake of contaminated fish or algal supplements [29,30]. Once ingested, the toxin is absorbed into the tissues, especially into hepatic tissue via the bile acid carrier system [1].

The current guidelines for safe exposure to the toxin in humans have been extrapolated from experiments performed in healthy animal models. In a 13-week study performed by Fawell et al., the No Observed Adverse Effect Level (NOAEL) was 40 μg/kg and the Low Observed Adverse Effect Level (LOAEL) was 200 μg/kg of body weight [1]. In accordance with these findings, the World Health Organization (WHO) established the provisional guideline value of 1 μg/L of MC-LR in drinking water. In 2015, as part of the Safe Drinking Water Act, the United States Environmental Protection Agency developed a Health Advisory for microcystins of 0.3 µg/L for bottle-fed infants and pre-school children and 1.6 µg/L for School-age children and adults [31]. Nevertheless, the effects of MC-LR in the setting of pre-existing liver disease remain unknown. 

Non-alcoholic Fatty Liver Disease (NAFLD) is one of the most common liver conditions in the United States. The incidence of NAFLD is increasing concurrent with the rise in obesity and diabetes [32,33,34]. NAFLD is characterized by the presence of steatosis/ inflammation with ≥ 5% fat infiltration into the liver and potentially elevated liver enzymes [35]. In the past two decades, the prevalence of this condition has nearly doubled, affecting about 20–30% of the population in the Western countries [36]. Given the increase in the occurrence of both NAFLD and cyanobacteria blooms globally, we evaluated the effects of MC-LR in the setting of pre-existing liver disease using the well-established Lepr^db^/J murine model of NAFLD [37]. Given NAFLD’s growing prevalence worldwide, several studies have recently investigated MC-LR’s effects in pre-existing NAFLD [18,28,38]. These studies have provided valuable insight, alerting us to the fact that individuals with pre-existing NAFLD may be more susceptible to MC-LR’s toxic effects. However, these studies utilize intraperitoneal (IP) and intravenous (IV) methods of MC-LR exposure. In our current study, we provide additional insight by using a robust genetic model of NAFLD and expose mice to MC-LR by gavage, an exposure route more relevant to environmental methods of exposure. We tested the hypothesis that chronic low dose oral exposure to MC-LR at levels below the NOAEL established in healthy animal models would exacerbate the hepatotoxicity seen in a NAFLD murine model. 

## 2. Results

### 2.1. Survival, Appearance and Weight

**Figure 1:**
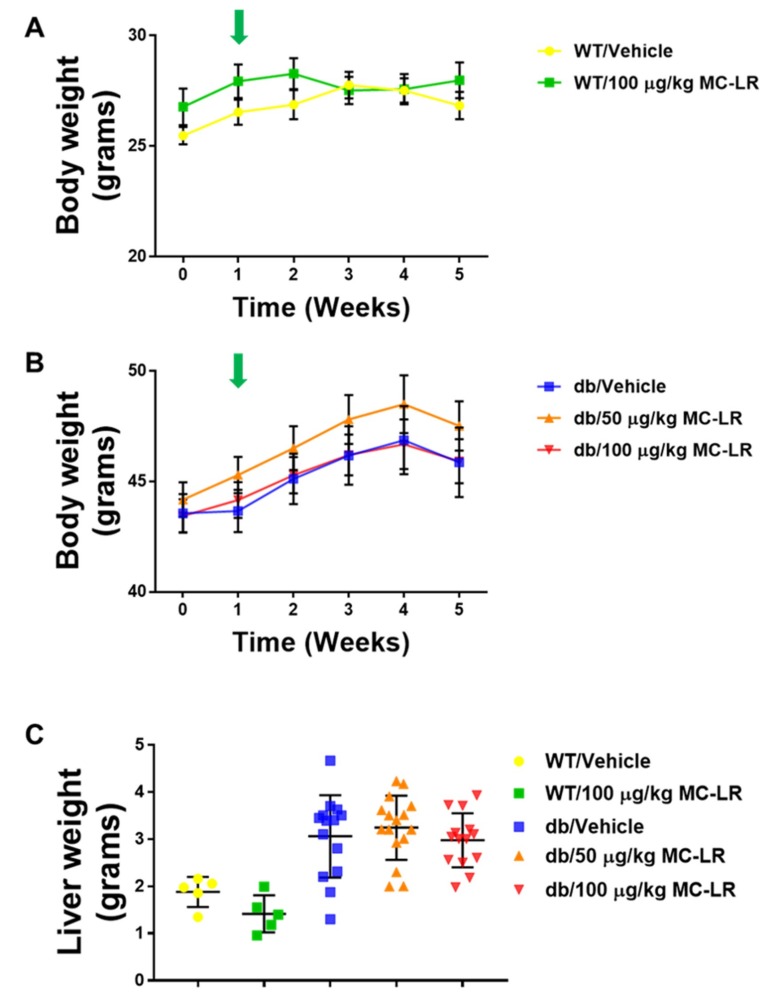
Effect of Microcystin-LR (MC-LR) on body and liver weights in both healthy (C57Bl/6J) and NAFLD (Lepr^db^/J) mice. (**A** and **B**) Total body weights. (A) C57Bl6/J (WT) mice exposed to vehicle (*n* = 5) or 100 µg/kg MC-LR (*n* = 5) and (B) Lepr^db^/J (db) mice exposed to vehicle (*n* = 13), 50 µg/kg MC-LR (*n* = 16), or 100 µg/kg MC-LR (*n* = 14). Mean and S.E.M. are indicated. (**C**) liver weights of animals from all groups. Individual, mean, and S.E.M. values indicated. NOTE: The green arrow over Week 1 represents the initiation of exposure to MC-LR or vehicle.

No visible symptoms of sickness or discomfort were observed in Lepr^db^/J mice from either the 50 μg/kg or 100 μg/kg MC-LR exposed groups. However, early mortality was observed in 1/17 mice (95% survival) from the 50 μg/kg MC-LR exposed group and 3/17 mice (83% survival) from the 100 μg/kg MC-LR exposed group (Kaplan-Meier log-rank *p* = 0.07, Figure S1A). In each case the animals died overnight and there was no observed acute trauma (e.g., tracheal rupture resulting in immediate death) or other signs of improper gavage technique such as visible signs of discomfort or bloating in the time preceding death although we were not able to fully exclude mortality attributable to the gavage procedure in these animals. It should be noted that the Lepr^db^/J mice are more susceptible to comorbidities including compromised immune system [39]. No deaths were observed in either the vehicle exposed Lepr^db^/J group (*n* = 13) or in either of the vehicle or 100 μg/kg MC-LR exposed healthy background strain control C57Bl/6J (Wild Type - WT) mice study groups (*n* = 5 mice/group each). The body weight gain throughout the exposure in Lepr^db^/J MC-LR exposed groups was not significantly different from the respective vehicle exposed group (Figure 1A,B). The physical appearance (Figure S1B–D) as well as the weight of the livers (Figure 1C) were not significantly different from the MC-LR-exposed groups in either the Lepr^db^/J or WT mice (data not shown). Other organs including heart, lung, and kidney were collected from both the Lepr^db^/J and C57Bl/6J control and MC-LR-exposed groups. There was no significant difference in organ weight between WT C57Bl/6J control and C57Bl/6J MC-LR-exposed mice as well as no significant differences among Lepr^db^/J vehicle control and Lepr^db^/J MC-LR exposed mice. (Tables S1 and S2).

### 2.2. Detection of MC-LR in Plasma and Urine

**Figure 2:**
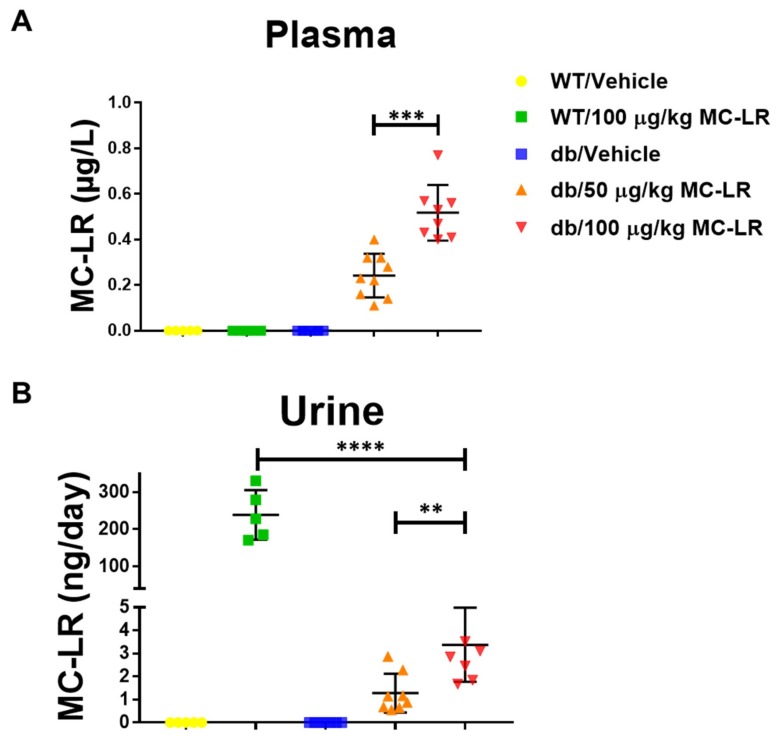
MC-LR determination in plasma and urine of both healthy (C57Bl/6J) and NAFLD (Lepr^db^/J) mice. (**A**) Plasma and (**B**) 24-h urine excretion levels of MC-LR in C57Bl/6J (WT) Leprdb/J (db) mice as assessed by Ultra-high-performance liquid chromatography coupled to triple quadrupole mass spectrometry (UHPLC-QqQ-MS/MS) and High-Performance Liquid chromatography–orbitrap mass spectrometry (HPLC-orbitrap-MS) at the end of the 4–week exposure protocol. For plasma analysis, WT/vehicle *n* = 5, WT/100 μg/kg MC-LR *n* = 5, db/vehicle *n* = 8, db/50 μg/kg MC-LR *n* = 9, db/100 μg/kg MC-LR *n* = 8. For 24-h urine excretion analysis, WT/vehicle *n* = 5, WT/100 μg/kg MC-LR *n* = 5, db/vehicle *n* = 7, db/50 μg/kg MC-LR *n* = 8, db/100 μg/kg MC-LR *n* = 8. The number of samples shown in the figure for the db mice vary due to insufficient amount of plasma or urine needed for the analysis. Significance calculated by unpaired Student’s t-test for the indicated comparisons. **, *p* < 0.01; ***, *p* < 0.001; ****, *p* < 0.0001.

To determine circulating levels and urinary excretion of MC-LR resulting from this exposure regimen, we collected plasma and 24-h urine samples at the end of the study prior to euthanasia. We then used Ultra-high-performance liquid chromatography coupled to triple quadrupole mass spectrometry (UHPLC-QqQ-MS/MS) and High-Performance Liquid chromatography–orbitrap mass spectrometry (HPLC-orbitrap-MS) to detect and quantify the MC-LR levels in animals which met the sample volume requirements for the analysis. MC-LR was detected in the plasma (Figure 2A) and urine (Figure 2B) of exposed Lepr^db^/J mice. Significantly more MC-LR was detected in the 100 µg/kg MC-LR exposed Lepr^db^/J mice (*p* < 0.001 for plasma and *p* < 0.01 for urine, respectively) as compared to those exposed to 50 µg/kg MC-LR. Interestingly, the toxin was undetectable in the plasma of C57Bl6/J mice exposed to the toxin (Figure 2A), whereas the amounts detected in the 24-h urine excretion samples were significantly elevated (nearly 100-fold) as compared to the Lepr^db^/J mice exposed to the same amount of the toxin (Figure 2B).

### 2.3. Blood Biochemistry

**Figure 3:**
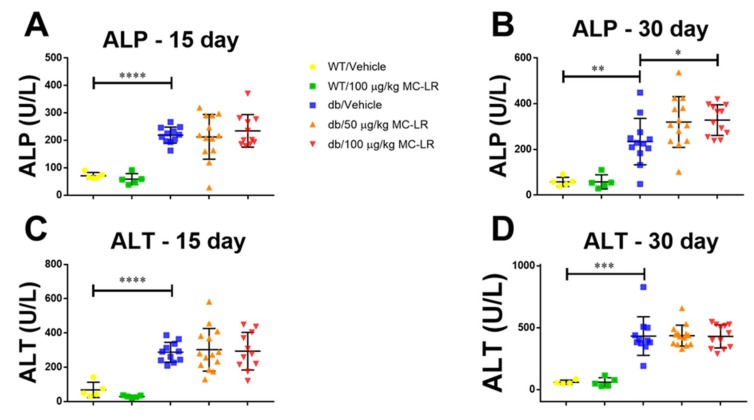
Effect of MC-LR on levels of liver injury enzymes in both healthy (C57Bl/6J) and NAFLD (Lepr^db^/J) mice. Circulating levels of Alkaline Phosphatase (ALP) (**A** and **B**) and Alanine aminotransferase (ALT) (**C** and **D**) were measured across all exposure groups at 15 days and 30 days after initial exposure. Significance between groups tested by unpaired Student’s t-test for indicated comparisons. For 15-day analysis, WT/vehicle *n* = 5, WT/100 μg/kg MC-LR *n* = 5, db/vehicle *n* = 11, db/50 μg/kg MC-LR *n* = 13, db/100 μg/kg MC-LR *n* = 11. For 30-day analysis, WT/vehicle *n* = 5, WT/100 μg/kg MC-LR *n* = 5, db/vehicle *n* = 12, db/50 μg/kg MC-LR *n* = 13, db/100 μg/kg MC-LR *n* = 12, *, *p* < 0.05, **, *p* < 0.01; ***, *p* < 0.001; ****, *p* < 0.0001.

To determine the levels of liver injury enzymes, namely alanine aminotransferase (ALT) and alkaline phosphatase (ALP), as well as to study the factors of blood biochemistry, we conducted a comprehensive diagnostic analysis using whole blood collected via retro-orbital bleed at days 15 and 30 during the exposure. Results from the comprehensive diagnostic analysis are shown in Tables S3 and S4. In paired analysis of the Lepr^db^/J mice, the levels of ALT were non-specifically elevated (15 vs. 30-day time points) in the vehicle exposed mice (*p* < 0.001), 50 μg/kg MC-LR exposed mice (*p* < 0.001), as well as the 100 μg/kg MC-LR exposed mice (*p* < 0.05, Figure S2A). On the other hand, the levels of ALP were only significantly elevated (15 vs. 30-day time points) in the 50 μg/kg MC-LR exposed mice (*p* < 0.05) as well as in the 100 μg/kg MC-LR exposed mice (*p* < 0.01), but not in the vehicle exposed mice (Figure S2B). In the unpaired, between group analysis (Figure 3), the only significant change in blood chemistry between vehicle and MC-LR exposed mice was that ALP levels were elevated in the 100 μg/kg MC-LR exposed mice vs. vehicle (*p* < 0.01) at the 30-day time point (Figure 3B). All other blood biochemistry markers did not show any significant change between vehicle or MC-LR exposed groups for either the Lepr^db^/J mice (Figure 3, Table S3) or the C57Bl/6J mice (Figure 3, Table S4).

### 2.4. Liver Histology

**Figure 4:**
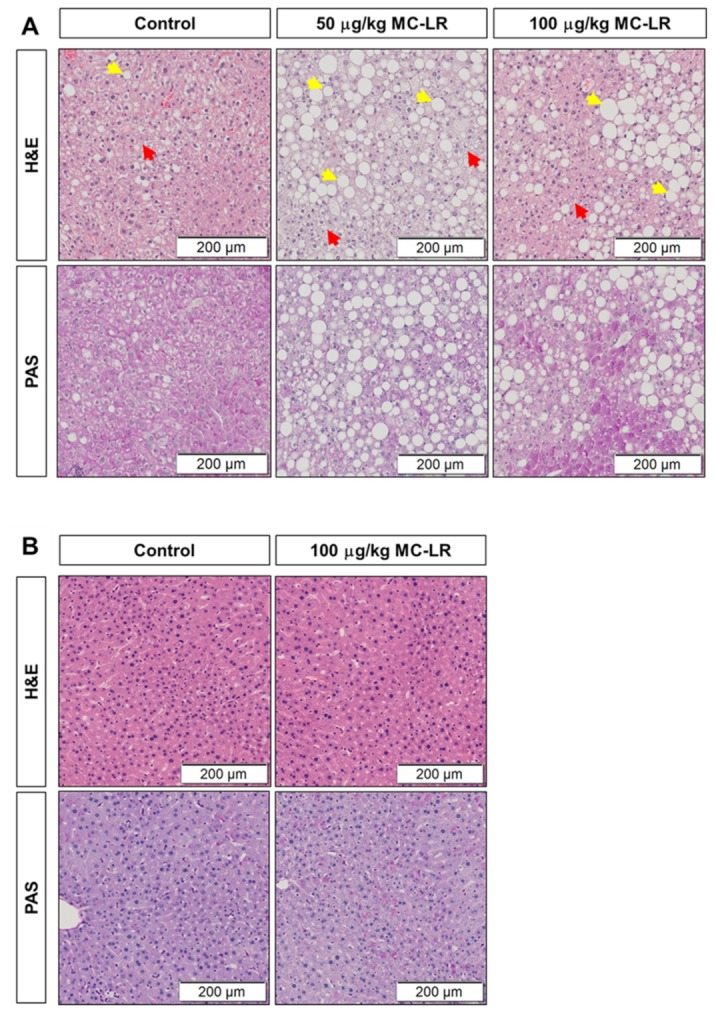

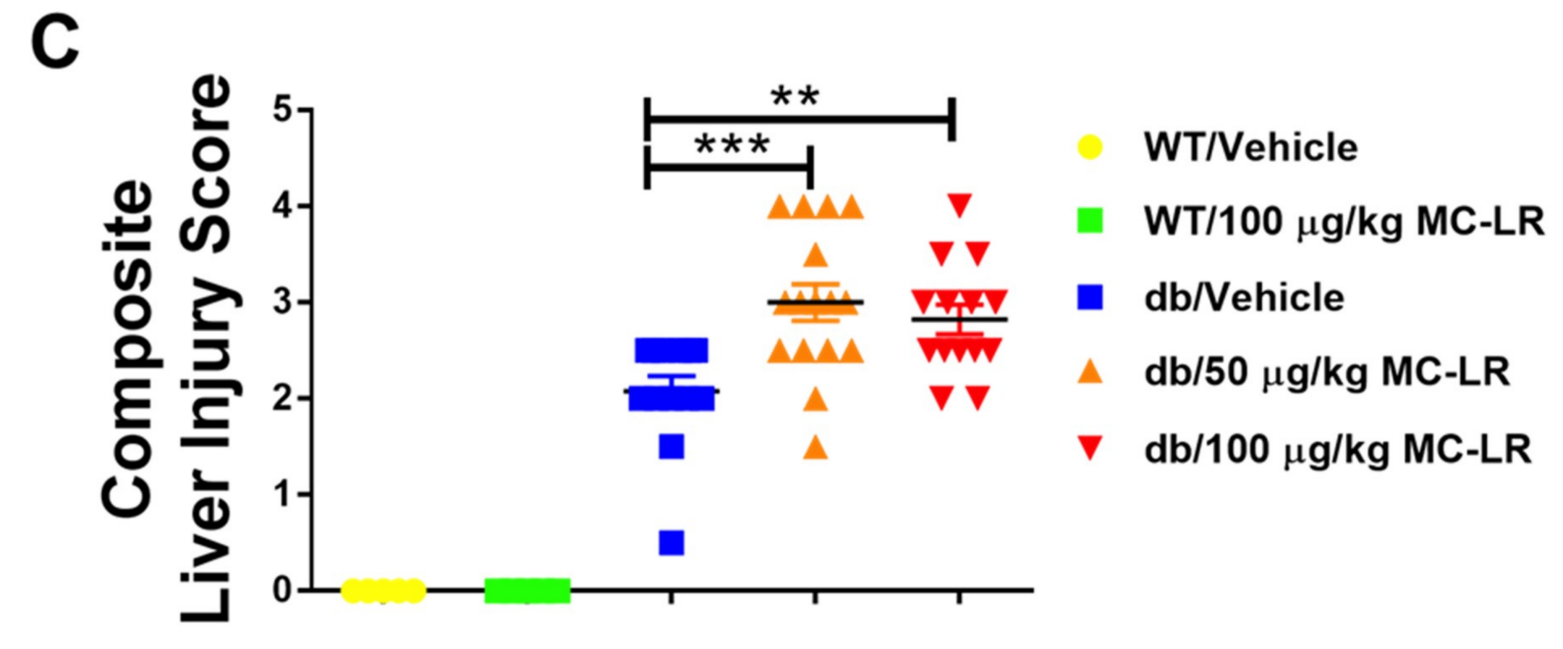
Histological analysis of hepatic injury in both healthy (C57Bl/6J) and NAFLD (Lepr^db^/J) mice. (A and B) Representative images of liver tissue sections from different exposure groups of Lepr^db^/J (db) mice (**A**) and C57Bl6/J (WT) mice (**B**) stained with Hematoxylin & Eosin (H&E) (top panels) and Periodic Acid-Schiff (PAS) (bottom panels) staining; yellow arrows indicate fat vacuoles, red arrows indicate fat infiltration inside hepatocytes; scale bar = 200 μm. (**C**) Composite liver injury score for C57Bl6/J mice (*n* = 5/group) and Lepr^db^/J mice receiving vehicle (*n* = 13), 50 μg/kg MC-LR (*n* = 16; ***, *p* < 0.0006 vs. vehicle) and 100 μg/kg MC-LR (*n* = 14; *, *p* = 0.0023 vs. vehicle) as determined by Kruskal–Wallis ANOVA and Mann–Whitney test. No significant injury was determined in either the vehicle or 100 μg/kg MC-LR exposed C57Bl6/J mice.

Histopathological analysis was performed using Hematoxylin & Eosin (H&E) and Periodic Acid-Schiff (PAS) staining of the liver sections. The slides were assessed by a pathologist, who was blinded to the group assignments, and histopathologic scoring was assessed based on the severity of fat infiltration, hepatic damage, glycogen content, and micro- and macro-vesicular lipid accumulation within the hepatocytes. As shown in Figure 4, significant hepatic injury was noted in both 50 μg/kg and 100 μg/kg MC-LR exposed Lepr^db^/J mice as compared to the vehicle control Lepr^db^/J mice. This was noted by the presence of fat vacuoles (Figure 4A, top panel, yellow arrows) as well as macro- and micro-vesicular fat infiltration and accumulation inside the hepatocytes (Figure 4A, top panels, red arrows). Furthermore, PAS staining demonstrated reduction in the glycogen content in Lepr^db^/J MC-LR exposed mice as compared to vehicle control, although this appeared to be more pronounced in the 50 μg/kg group (Figure 4A, bottom panels). Based on histopathologic scoring, liver injury was significantly increased in MC-LR exposed Lepr^db^/J mice as compared to the vehicle control Lepr^db^/J mice (*p* < 0.001 in 50 μg/kg and *p* < 0.05 in 100 μg/kg, Figure 4C). However, no significant changes were observed in the liver sections of MC-LR exposed C57Bl/6J mice compared to vehicle control C57Bl/6J mice (Figure 4B,C). The H&E and PAS derived injury scores for each individual animal are presented in Table S5.

### 2.5. Genetic Analysis Hepatotoxicity and Oxidative Stress

Since micro-vesicle lipid accumulation is associated with hepatic inflammation and oxidative stress, we assessed markers of inflammation and oxidative stress in the livers of Lepr^db^/J mice after microcystin exposure. Interestingly, we observed that several genetic markers associated with hepatotoxicity and oxidative stress response were significantly upregulated as compared to the respective db/vehicle control (Tables S6 and S7). Significantly (*p* < 0.05) upregulated markers of hepatotoxicity (*Cxcl12,* 170.3 fold upregulated; *Casp3*, 15.6 fold upregulated; *Cyp1a2*, 187.2 fold upregulated; *Rb1*, 2.5 fold upregulated), necrosis (*Fam214a*, 15.6 fold upregulated; *Mlxip1*, 97.1 fold upregulated; *Col4a1*, 33.2 fold upregulated), nongenotoxic hepatocarcinogenicity (*Ccng1*, 19.3 fold upregulated) as well as oxidative stress response (*Prdx2*, 2876.3 fold upregulated; *Gpx5*, 4.7 fold upregulated) were noted in the 50 μg/kg MC-LR exposed db mice vs. db/vehicle controls. Limited analysis of targeted genes associated with hepatotoxicity (*Cyp1a2*, *p* = 0.7; *Slc17a3*, *p* = 0.9), necrosis (*OSMR*, *p* = 0.9), steatosis (*CD36*, *p* = 0.5), phospholipidosis (*Serpina3n*, *p* = 0.07) and cholestasis (*Abcb4*, *p* = 0.6) were tested by RT-PCR in the C57Bl/6J groups and were not found to have any change in the gene expression between vehicle and MC-LR exposed groups. 

### 2.6. Phosphoproteomic Analysis

Based on the above results and the fact that MC-LR is a known protein phosphatase inhibitor, we next examined phosphoproteomic pathways, which were affected in the livers of MC-LR exposed Lepr^db^/J mice, to better understand the possible disruptions in signaling pathways associated with the hepatotoxicity in this model. Phosphoproteomic analysis with mass spectrometry-based label-free quantification of TiO_2_ enriched samples was used to determine the phosphorylation sites and signaling pathways affected by MC-LR exposure.

Data analysis using Maxquant identified 9616 phosphorylation sites in total. A moderated t-test indicated that 10 phosphorylation sites were affected by 50 µg/kg microcystin exposure compared to vehicle control samples (q < 0.1, *n* = 8, Table 1, Figure S3A,B). Similar comparison was done, to indicate changes in phosphorylation sites, between 100 μg/kg microcystin exposure and Vehicle control samples Table 1, Figure S3C,D). An alternative analysis using linear regression of site intensity vs microcystin dose identified 25 sites with a linear relationship between dose and intensity (*q* < 0.1, *n* = 7, Table 1).

Enrichment analysis of Gene Ontology (GO) biological processes, using Platform for Integrative Analysis of Omics (PIANO, v2.0.2., Bioconductor, Package for R, Sweden) software [40], was used to identify molecular pathways that were affected by microcystin exposure. Here we tested whether the mean t-statistic for each pathway was different from 0 indicating that the process components taken together were affected by MC-LR exposure. Exposure to 50 µg/kg MC-LR resulted in decreased phosphosite abundance of 91 pathways which represented 16 biological processes including urogenital system development, regulation of T cell proliferation and response to oxidative stress as well as positive regulation of cell cycle proliferation. The 16 processes selected to reduce redundancy are shown in Table 2. No GO processes were hyperphosphorylated in response to 50 µg/kg MC-LR (PIANO, FDR < 0.1). An analysis to identify specific classes of kinases that were affected by microcystin exposure failed to identify any significant kinase enrichment (PIANO, FDR < 0.1). Pathways in the Reactome pathway database [41] were also tested for changes in response to microcystin exposure. All GO and Reactome results are shown in Tables S8–S10. 

In order to identify which microcystin-affected systems were the most sensitive to MC-LR exposure, fuzzy c-means clustering was used to identify common phosphorylation site abundance profiles across the MC-LR exposure groups. Among them, 4259 sites were assigned to one of 6 clusters (Figure S4). Biological process categories that were identified as affected by 50 µg/kg microcystin at an FDR < 0.03 were tested for enrichment in the c-means clusters using a Fisher’s exact test. The results of this enrichment analysis demonstrated significant enrichment of 11 biological processes (Fishers exact test, *p* < 0.02) including several processes related to immune cell function as well as kidney/urogenital system development (Table 3). Cluster 3 contains sites that were dephosphorylated in both the 50 and 100 μg/kg samples compared to controls. It had the greatest number of enriched categories including several processes related to immune cell function as well as kidney/urogenital system development, suggesting that proteins in those categories are dephosphorylated by MC-LR exposure. Equivalent analysis using pathways that were affected by 100 µg/kg microcystin (FDR < 0.03) did not identify any cluster-enriched pathways. Cluster enrichment of Reactome pathways were also conducted as previously described [41] and are shown in Tables S8–S10. Analysis using the UniProt database was performed to summarize all the proteins with their phosphorylation sites affected by either 50 or 100 μg/kg MC-LR exposure (Table S11).

## 3. Discussion

In the current study, we used Lepr^db^/J mice, a well-established genetic model for NAFLD [42], to examine the effect of prolonged low dose oral exposure to MC-LR in mice with pre-existing liver disease. The dosages used in the current study approximated the 40 μg/kg NOAEL [1] albeit with a reduced dosing regimen (every other day vs. daily) and total study duration (4 weeks vs. 13 weeks) [1,2]. This chronic low dose regimen resulted in significant increases in both circulating plasma levels of MC-LR as well as increased 24 h urinary excretion of MC-LR in the Lepr^db^/J mice, as measured by UHPLC-QqQ-MS/MS and HPLC-orbitrap MS as we have described [43]. Interestingly, while there was no mortality or clinically observable MC-LR induced liver injury in the non-NALFD wild type C57Bl/6J group in our study, we did note a decreasing, albeit non-significant trend in survival in the MC-LR exposed Lepr^db^/J mice, despite similar body and liver weights, as well as the gross morphology of the livers measured in the surviving mice. Additionally, non-NALFD wild type C57Bl/6J had undetectable circulating plasma levels of MC-LR and a highly significant increase in the 24-h urine excretion of the toxin as compared to the Lepr^db^/J mice. This finding may be an indication of differing metabolic and excretory mechanisms between the healthy vs. NALFD diseased models and is likely relevant to the increased pathology and gene expression associated with hepatotoxicity and oxidative stress in the Lepr^db^/J mice.

Similarly, we noted in the Lepr^db^/J NAFLD model that while ALT levels were elevated non-specifically, ALP levels had a modest but significant increase specifically in the MC-LR exposed Lepr^db^/J mice. Neither ALT or ALP levels were significantly elevated in the wild type C57Bl/6J mice exposed to MC-LR at the highest dose studied, 100 μg/kg. While ALT is a marker of both acute or chronic liver injury, ALP is a marker of cholestatic predominance [44]. This indicates that ALT may not be a clinically useful marker of MC-LR induced liver injury in the setting of NAFLD. On the other hand, the fact that ALP levels were significantly elevated in MC-LR exposed Lepr^db^/J mice suggests that MC-LR may exacerbate liver damage in a cholestatic and obstructive pattern.

There are different patterns of hepatic-injury including hepatocyte-degeneration, intracellular fat accumulation, apoptosis, inflammation, regeneration and fibrosis [45]. Indeed, our histological analysis confirmed that hepatic injury in the form of hepatic micro-vesicular lipid accumulation, ballooned hepatocytes and reduced glycogen content was significantly higher in the MC-LR exposed Lepr^db^/J mice. This finding indicates that exposure to MC-LR affects lipid metabolism and leads to increased fat accumulation in the livers of NAFLD mice and supports the observation that MC-LR exposure may promote cholestasis in this setting. Recent studies have investigated MC-LR toxicity in the setting of pre-existing NAFLD [18,28,38]. Clarke et al. studied the acute effects of MC-LR by a single-dose intravenous (IV) injection (20 μg/kg) and a single-dose of intraperitoneal (IP) injection (60 μg/kg) in two separate rat models of NAFLD: a methionine and choline deficient (MCD) diet and a high fat/high cholesterol (HFHC) diet [18]. Observed findings were informative but different between the two NAFLD models. While plasma MC-LR area under the concentration-time curve (AUC) was doubled and biliary clearance (Cl_bil_) was unchanged in the MCD rats, AUC was unchanged and CL_bil_ was doubled in the HFHC rats. In addition, while liver pathology decreased in the MCD rats due to decreased binding to PP2A, hepatic inflammation, plasma cholesterol, proteinuria, and urinary KIM1 were increased in HFHC rats. These results reveal that pre-existing NAFLD potentially affects subject susceptibility to MC-LR toxicity, but there is variation to exact manifestations due to differences in NAFLD induction. Our current study provides a robust genetic model of NAFLD in mice that helps further the investigation of MC-LR toxicity in pre-existing NAFLD. In addition, our study provides a chronic exposure model by gavage, a more physiologically relevant method of exposure, mimicking a more typical exposure route in animals and humans. 

Other studies have investigated the potential of NOX2 as a mediator of MC-LR toxicity in the setting of pre-existing NAFLD [28,38]. Albadrani et al. have shown that in mice with pre-existing NAFLD, intraperitonial exposure of MC-LR potentially activates hepatic Kupffer cells and stellate cells through the NOX2 pathway, causing an exacerbation of pre-existing liver pathology through increased CD68 and increased pro-inflammatory cytokines. Sarkar et al. has shown that intraperitonial exposure of MC-LR in NAFLD mice alters the gut microbiome, leading to inflammatory processes within the intestines. Again, while these studies have provided invaluable insight using intraperitoneal-delivered MC-LR in diet induced NAFLD mice, our study builds upon these results by utilizing a genetic model of NAFLD with a physiologically relevant oral exposure of MC-LR.

MC-LR is a potent inhibitor of protein phosphatases 1 (PP1) and 2A (PP2A), which are responsible for dephosphorylating serine and threonine residues on regulatory and structural proteins. The inhibition of PP1 and PP2A leads to increased protein phosphorylation, which may alter the structure of the cell cytoskeleton [46]. It has been demonstrated that oxidative stress may contribute to the toxicity of MC-LR and that the formation of excessive free radical species from oxidative lipid alterations causes an increase in lipid peroxidation in mice serum [11,46]. Since PP2A regulates several mitogen-activated protein kinases (MAPKs), its inhibition by MC-LR may have secondary effects on downstream MAPK signaling pathways involved in essential cellular processes [47]. Additionally, many inflammatory and immune responses are regulated by several MAPK signaling pathways, making the release of pro-inflammatory cytokines a consequence of MAPK disruptions caused by MC-LR [48].

Our genetic and phosphoproteomic analysis of livers from MC-LR exposed NAFLD mice is in agreement with the aforementioned studies. In the present study, we observed that genes associated with hepatotoxicity (*Cxcl12, Casp3, Cyp1a2, Rb1*), necrosis (*Fam214a, Mlxipl, Col4a1*) and nongenotoxic hepatocarcinogenicity (*Ccng1*) were significantly upregulated in the mice that were exposed to MC-LR. Oxidative stress is another mechanism associated with MC-LR toxicity [49]. Many in vitro studies have shown that exposure to MC-LR can cause oxidative stress in cells, further leading to apoptosis [50,51,52,53]. Our study shows that exposure to MC-LR significantly upregulated the expression of oxidative stress response genes such as *Prdx2 and Gpx5*. As mentioned earlier, limited analysis of targeted genes associated with hepatotoxicity (*Cyp1a2*, *Slc17a3*), necrosis (*OSMR*), steatosis (*CD36*), phospholipidosis (*Serpina3n*) and cholestasis (*Abcb4*) were tested for the C57Bl/6J groups and were not found to show any change in gene expression between vehicle and MC-LR exposed groups.

Our phosphoproteomic analysis revealed that MC-LR exposure significantly affected Reactome pathways related to immune function, urogenital system development, T cell proliferation, cellular response to oxidative stress, and cell cycle regulation pathways. Studies have shown that MC-LR exposure in mice increases the levels of superoxide dismutase and catalase as well as increases the expression of *TNF-α, NFκB, IL-6* and *IL-1β* in HepG2 cells exposed with MC-LR [53,54]. Another study showed an increase in transcription of IL-8, a chemokine involved in the recruitment of monocytes and neutrophils during inflammation, in fish that were exposed to MC-LR [55]. In the present study MC-LR exposure regulated pathways related to Toll-like receptor (TLR) 2 and 6 signaling as well as those related to cell cycle and cell division regulatory functions. TLR2 activation has been shown to activate the PI3K/AKT/NF-kB pathway in immunological response to MC-LR in testicular cells [56]. These studies performed in various in vivo and in vitro models, complement our findings about the various pathways that are affected by exposure with MC-LR. 

The fact that we did not note significant liver injury in the C57Bl/6J mice in the current study is in contrast with other studies where damage to mice was observed after oral exposure of MC-LR at similar doses of MC-LR by Zhang et al. and He et al. [6,57]. However, it should be noted that Zhang et al. gavaged the mice every 24 h for 28 days while in our study the mice were only gavaged every 48 h. Therefore, the total dosing in these mice was doubled compared to our study and this may account for the differences observed. In the He et al. study, the researchers used the Balb/c strain, whereas we used the C57Bl/6J because this strain is a better background control for the Leprdb/J mice. Importantly strain differences exist between Balb/c and C57Bl/6J strains where Balb/c mice have been shown to be more susceptible and C57Bl/6J mice less susceptible to hepatic injury [58,59]. 

MC-LR has been observed in freshwater environments around the world at varying levels. In the USA, 1000 μg/L of MC-LR was detected in the Lake Erie region [60]. In August 2014, elevated microcystin levels in the US city of Toledo, Ohio, led to a drinking water crisis when the city was left without drinking water for >2 days [61]. MC-LR not only accumulates within the water, but also accumulates within the aquatic ecosystem as extensively reviewed by Pham et al. [62]. MC-LR within the aquatic food chain, as well as within aquatic environments, pose potential risk of exposure in humans. In fact, human cases of exposure have been extensively reviewed by Svircev et al. [63]. A study in Australia reported MC-LR levels measured at 1 and 12 μg/L on two separate occasions within recreational locations (lakes and rivers in southern Queensland and in the Myall Lakes area of New South Wales), with surveyed individuals reporting gastrointestinal and respiratory symptoms [64]. In China, levels of MC-LR in Lake Chaohu have been reported to range from 2.2–3.9 μg/L [29], with measurable liver damage found in fishermen that work on the lake. In Sweden, 121 individuals from three villages reported abdominal pain, nausea, vomiting, diarrhea, fever, headaches, and muscle pain after their drinking water became discolored [63]. It was later found that the raw lake water contained 1 μg/L of MC-LR.

Taken together, our study provides insight into the potential increase in susceptibility of individuals with pre-existing liver conditions such as NAFLD, to the harmful effects of MC-LR exposure. By using exposure levels below the established NOAEL value of 40 μg/kg, our study reveals that MC-LR can exacerbate the liver damage in the setting of pre-existing liver disease. Such liver damage can be attributed to excess micro-vesicular lipid accumulation in the liver, upregulation of genes associated with hepatotoxicity and oxidative stress as well as changes in phosphorylation of the biological pathways related to cell cycle and immune response regulation. The results of this study suggest a need to review the preventative guidelines for safe exposure to MC-LR in at-risk settings, such as those with a pre-existing NAFLD. Indeed, we have recently demonstrated that MC-LR not only prolongs, but also worsens the severity of pre-existing colitis, supporting the notion that the effects of cyanotoxins such as MC-LR may be magnified in certain at-risk populations [65]. We have also demonstrated that the levels of the cyanotoxin can be detected in both plasma and urine samples of mice that were gavaged with low doses of the toxin, using UHPLC-QqQ-MS/MS and HPLC-orbitrap-MS methods [43]. This suggests that mass spectrometric analysis can be used in a clinical setting to assess the exposure to the cyanotoxins in humans. Further studies are warranted to examine the mechanistic basis of MC-LR hepatotoxicity in conditions such as NAFLD in order to assess diagnostic and therapeutic strategies for these potentially vulnerable populations. Additionally, epidemiological observations are needed to provide clinical context for the potential effects of cyanotoxins such as MC-LR in vulnerable patient populations.

### Limitations 

An important limitation of the current study was the fact that, because of the premature deaths in the MC-LR exposed NAFLD groups, not all animals were available for histopathological, biochemical, and phosphoproteomic examination. This may have affected the significance and dose dependence of the histopathological, biochemical, and phosphoproteomic changes we reported. It should also be noted that the doses used in this study are likely above doses to which humans, including those with NALFD, are exposed. Another limitation of the current study is that we were not able to measure MC-LR levels in the livers of the exposed mice nor did we perform analysis of activities or expression of PP1 and PP2A. However, despite these limitations, our study is the first to report the effects of chronic low dose oral exposure to MC-LR in a model of NAFLD. Our study suggests that in settings of pre-existing liver disease such as NAFLD, there may be an increase in the susceptibility to cyanotoxin toxicity.

## 4. Materials and Methods

### 4.1. Mice

Eight-week-old male B6.BKS(D)-Lepr^db^/J (JAX Stock No. 000697, B6 db) mice (hereafter referred to as Lepr^db^/J mice) on the C57Bl/6J background and wild type C57Bl/6J (JAX Stock No. 000664, Black 6) healthy background strain control mice were obtained from The Jackson Laboratory (Bar Harbor, Maine, USA) and maintained in the Department of Laboratory Animal Research at University of Toledo. All the mice were specific-pathogen free status and were housed in plastic cages (five mice per cage) and fed ad libitum on balanced rodent diet (Teklad global 16% protein diet, Envigo, Indianapolis, IN, USA) and water. The mice were kept in a well-ventilated room maintained at 23 ± 1 °C on a 12-h light and dark cycle. The mice were allowed to acclimatize for a week, before the beginning of the study. All the protocols were approved by the University of Toledo Institutional Animal Care and Use Committee (IACUC protocol number 108663, approval date February 9, 2016) and conducted in accordance with the National Institutes of Health (NIH) Guide for the Care and Use of Laboratory Animals.

### 4.2. Exposure and Experimental Design

**Figure 5:**
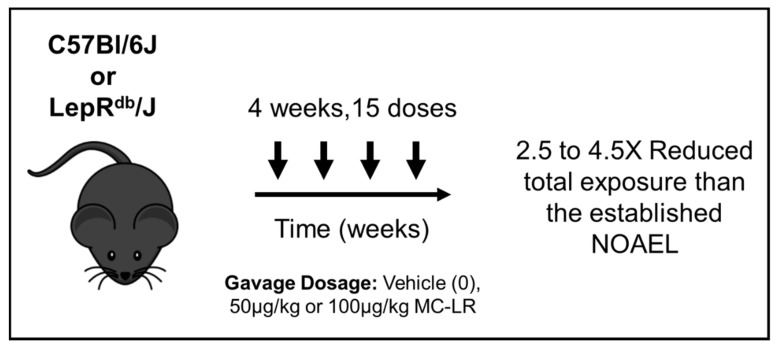
Schematic representation of the study design used to study the effect of low doses of MC-LR on healthy C57Bl/6J and NAFLD LepR^db^/J mice. Ten-week-old mice were exposed with vehicle, 50 μg/kg or 100 μg/kg of MC-LR for a period of 4 weeks (15 total administrations) every 48 h. The mice in the vehicle control group were exposed with an equivalent volume of purified water.

At 10-weeks of age, Lepr^db^/J mice were randomly divided into three groups with 13–17 mice per group (Figure 5). Group 1 was the Lepr^db^/J vehicle control group which was exposed to water with volume equivalent to that of the MC-LR exposed mice (300 μL). Group 2 and 3 consisted of Lepr^db^/J mice that were exposed to 50 μg/kg and 100 μg/kg of body weight of MC-LR (Item No. 10007188, Cayman Chemicals, Ann Arbor, MI, USA) respectively. MC-LR or vehicle control was administered to the mice via gavage every 48 h for a period of 4 weeks (15 total administrations over 4 weeks). These doses approximate the currently accepted NOAEL (40 μg/kg) established over a 13 week study [1]. The toxin was freshly prepared by dissolving it in purified water at a working concentration of 0.5 mg/mL. The mice were weighed once every week. Blood samples were collected at day 15 (midpoint) and 30 (endpoint) via retro-orbital bleeding (after isoflurane anesthesia) for analyzing blood chemistry, and at study termination, via intracardiac puncture after euthanasia. At the end of the study, mice were placed in metabolic cages for a 24-h urine collection immediately following the final dose of MC-LR or vehicle. 24-h urine samples were collected and stored at −80 °C. Mice were then removed from metabolic cages and euthanized for final blood and organ collection after another 24 h (i.e., a total of 48 h after last MC-LR/vehicle gavage). Blood samples were collected at day 15 (midpoint) and 30 (endpoint) via retro-orbital bleeding (after isoflurane anesthesia) for analyzing blood chemistry and at study termination via intracardiac puncture after euthanasia.

The animals were humanely euthanized and flushed with 10 ml of 1X Phosphate Buffered Saline (PBS) (Fisher Scientific, Pittsburgh, PA, USA). Liver tissues from individual animals were collected and weighed. Part of the liver tissue was frozen in OCT embedding media (Fisher Scientific, Pittsburgh, PA, USA) and another section was fixed with 4% *w/v* Formalin (Fisher Scientific, Pittsburgh, PA, USA) for histological purposes. The remainder of the tissue was snap frozen in liquid nitrogen and then stored at −80 °C until further use for experimental purposes. In a parallel study, wild type C57Bl/6J mice were randomly divided into 2 groups: a vehicle control and 100 μg/kg MC-LR exposed group, which were exposed as detailed above.

### 4.3. Histological Studies

Liver sections were collected and immediately fixed in 4% formalin solution for 24 h, dehydrated in 70% ethanol, embedded in a paraffin block, and cut with a microtome to yield sections of 4-micron thickness. Hematoxylin & Eosin (H&E) and Periodic Acid-Schiff (PAS) staining were then performed on these sections. Whole slides were scored for hepatic injury by a pathologist, who was blinded to the study group assignments. Hepatic injury was assessed in both H&E and PAS was scored on a semi-quantitative scale of 0 (none or least hepatic injury) to 4 (maximum hepatic injury) and a composite injury score was taken as the average of the H&E and PAS injury score for each liver. Hepatic injury was assessed according to established methods including pale coloration of the tissue, infiltration of microvesicular lipid accumulation inside the hepatocytes, reduced glycogen content as well as ballooned hepatocytes with marked macro- and/or micro-vesicular lipid infiltration [45]. Hepatic damage was characterized by pale coloration of the tissue, infiltration of microvesicular lipid accumulation inside the hepatocytes, reduced glycogen content as well as ballooned hepatocytes with marked macro- and micro-vesicular lipid infiltration. A detailed description of the observations can be found in Table S5.

### 4.4. Blood Chemistry

Blood was collected via retro-orbital bleed in heparinized Micro-Hematocrit Capillary tubes (Cat no. 22-362-566, Fisher Scientific, Pittsburgh, PA, USA). Whole blood was then loaded onto Abaxis rotor with VetScan2 Chemistry Analyzer (Ref: 500-0038, Abaxis, Union City, CA, USA) with a comprehensive diagnostic profile for the quantitative analysis of Alanine aminotransferase (ALT), albumin (ALB), alkaline phosphatase (ALP), amylase (AMY), globulin (GLOB), glucose (GLU), blood urea nitrogen (BUN) and total protein (TP). 

### 4.5. MC-LR Determination in Plasma and Urine

Both plasma and 24-h urine samples were collected post MC-LR exposure. Blood was collected by cardiac puncture in K3-EDTA microtubes (Sarstedt, Newton, NC, USA) and separated plasma was stored at −80 °C until further use. MC-LR in urine samples was quantified using both Ultra High Pressure Liquid Chromatography–triple Quadrupole–tandem Mass Spectrometry (UHPLC-QqQ-MS/MS, Columbia, MD, USA) and High Pressure Liquid Chromatography (Shimadzu Technologies, Addison, IL USA)–Orbitrap Fusion Mass Spectrometry (Thermo Fisher Scientific, San Jose, CA, USA) system, while plasma samples were analyzed using HPLC-Orbitrap Fusion MS as we have previously described [43].

### 4.6. Genetic Analysis of Hepatotoxicity and Oxidative Stress

For genetic analysis, each exposure group shows data from 3 arrays (i.e., biological triplicates) and each array used a pooled cDNA sample from 4 mice/group. Hence, the data is representative of 12 mice from each exposure group. The data was analyzed using Qiagen analysis software (Qiagen, Germantown, MD, USA) and the criteria for fold change is based on any changes that are at least 2-fold above the normalized value for the vehicle exposed group. The *p* value for significance was considered as *p* < 0.05. RNA was extracted from liver tissues and isolated using the QIAzol/Chloroform extraction method. Approximately 500 ng of extracted RNA was used to synthesize cDNA (QIAGEN’s RT2 First Strand Kit (Qiagen, Germantown, MD, USA). The cDNA was then used to run RT^2^ Profiler^TM^ Mouse Hepatotoxicity Quantitative PCR Array (Cat No. PAMM-093Z, Qiagen, Germantown, MD, USA) as well as RT^2^ Profiler^TM^ Mouse Oxidative Stress and Antioxidant Defense Quantitative PCR Array (Cat No. PAMM-065Z, Qiagen, Germantown, MD, USA) performed utilizing a QIAGEN Rotor-Gene Q thermo-cycler. Because previous reports have shown hepatotoxic and oxidative stress mediated injury induced by MC-LR, we selected targeted arrays to assess several measures of hepatotoxicity using the RT^2^ Profiler^TM^ Mouse Hepatotoxicity Quantitative PCR Array (Cat No. PAMM-093Z which assesses genetic markers of Hepatotoxicity—Cxcl12, Cyp1a2, Casp3, Rb1; Nongenotoxic hepatocarcinogenicity—Ccng1; Necrosis—Fam214a, Mlxipl; Steatosis—CD36 and Cholestasis—Abcb1A, Abcb4) as well as RT^2^ Profiler^TM^ Mouse Oxidative Stress and Antioxidant Defense Quantitative PCR Array (Cat No. PAMM-065Z which assesses genetic markers of peroxiredoxins; glutathione peroxidases; superoxide dismutases; oxygen transporters and oxidative stress response). Both arrays use Actin b, β2-microglobulin, GAPDH, Gusb and Hsp90ab1 as the housekeeping genes. All RT-qPCR was performed utilizing a QIAGEN Rotor-Gene Q thermo-cycler.

### 4.7. Proteomic Analyses of TiO_2_ Enriched Phosphopeptides

Sample Preparation: Mouse liver tissue was homogenized in mass spectrometry grade water using a Potter Elvehjem Teflon on glass tissue homogenizer kept on ice. Homogenates were brought to 2% lithium dodecyl sulfate (LiDS) then heated to 95 °C for 5 minutes. Heat inactivated samples were exposed with 5 mM dithiothreitol (DTT) to reduce disulfide bonds, alkylated with 15 mM iodoacetamide (IAA) and then 5 mM additional DTT was added to quench the remaining IAA. Proteins were precipitated and LiDS removed by addition of 5 volumes of methanol. Methanol-washed pellets were resuspended in 0.5% deoxycholate (DOC) in 1X phosphate buffered saline plus 40 mM TEAB using sonication delivered by a QSonica cup-horn sonicator as necessary. Protein concentrations in the DOC solubilized samples were determined using a Bradford assay then 0.5 mg of protein was trypsinized using an overnight incubation with 2 μg Promega sequencing grade trypsin. Digested samples were centrifuged to remove particulates and the entire sample dried by speed-vac then resolubilized in 100 μL of 65% acetonitrile, 2% trifluoroacetic acid (TFA) and at 25% saturation with glutamic acid. Phosphopeptides were selected from the digests on an AssayMap Bravo (Agilent Technologies, Santa Clara, CA, USA) robot using TiO_2_ cartridges. Phosphopeptides were analyzed at the Wayne State Proteomics Core using LC-MS/MS on an Orbitrap Fusion MS system (Thermo Fisher Scientific, San Jose, CA, USA). Each sample was analyzed independently using reversed-phase chromatography on an Acclaim PepMap RSLC (Thermo Fisher Scientific, San Jose, CA, USA), 75 µm × 25 cm column (Dionex, Sunnyvale, CA, USA). Peptides were eluted from the column in a 2-h gradient from 5% to 30% acetonitrile and analyzed directly by MS/MS using the Orbitrap Fusion.

### 4.8. Proteomic Data Analysis

MS spectra were searched against the Uniprot mouse complete database downloaded on 14 July 2017 (16,884 entries) using MaxQuant v1.6.2.10. (Max Planck Institute, Munich, Germany) [66]. Phosphorylation at Serine, Threonine and Tyrosine residues was set as a variable modification and the default penalty for modified peptide identification was reduced so that a minimum score of 20 and a minimum delta score of 3 were required for modified peptides. Match between runs was enabled. All other parameters were left at their default values. All analyses except the kinase enrichment analysis used phosphorylation sites without regard to localization confidence. Kinase enrichment analysis used all sites localized with > 80% confidence by Maxquant. Subsequent analysis used R v3.4.3 (http://www.R-project.org/) (The R Foundation, Vienna, Austria).

Phosphorylation site abundances were normalized so that each sample had the same median. Differentially abundant sites were identified using a moderated t-test [67]. A q-value was calculated for each site to account for multiple testing [68]. Gene Ontology (GO) biological processes that were affected by exposure were identified using Platform for Integrative Analysis of Omics Data (PIANO) [40]. T-statistics from the moderated t-test were submitted to PIANO and pathway enrichment was determined using the t-statistic mean. Phosphoproteins with multiple sites were not summarized and each site was submitted to PIANO individually. PIANO uses a permutation test to calculate an FDR corrected *p*-value. To reduce redundancy due to multiple GO categories with similar membership, affected pathways were clustered by phosphoprotein membership similarity and only the pathway with the lowest *p*-value in each cluster was reported. Pathways clustering was done using dynamic tree cut [69]. Pathway overlap was calculated as: (number of sites common to both pathways/number of sites in the smaller pathway). Kinase analysis was carried out using the regular expression-type kinase motifs distributed with Perseus software [70]. Identified phosphorylation sites that matched kinase criteria were converted to PIANO “kinase sets” and each kinase was tested for enrichment by PIANO analysis as above. Fuzzy c-means clustering was used to identify dose-response patterns in the data [71]. Sites were considered members of a cluster if they had greater than 0.5 membership. The number of clusters was set to 6 based on inspection of a plot of the number of sites clustered vs. the number of clusters. All sites were submitted to clustering without regard to their statistical significance. Gene Ontology (GO) biological processes were tested for enrichment in dose-response clusters using Fisher’s exact test for an increased frequency of pathway components in cluster members versus cluster non-members. As for the samples from mice exposed with 100 μg/kg of MC-LR, Reactome pathways were tested for enrichment in dose-response clusters using Fisher’s exact test for an increased frequency of pathway components in cluster members versus cluster non-members.

### 4.9. Statistical Analysis

Statistical analysis of all non-proteomic data was done using GraphPad PRISM 7 software (San Diego, CA, USA) and comparison within groups was done using Unpaired Student’s t-test and Analysis of Variance (ANOVA) with Dunnette’s multiple comparisons test. All data are presented as mean ± standard error of the mean (SEM) and a *p*-value of < 0.05 was considered to be statistically significant.

## Figures and Tables

**Table 1:** Effect of microcystin on phosphorylation sites in Lepr^db^/J liver protein. Number of sites affected by microcystin exposure in Lepr^db^/J livers using moderated t-tests or linear regression. Results were corrected for multiple testing, *n* = 8 for 50 µg/kg and *n* = 7 for 100 µg/kg microcystin.

Test	*q* < 0.10	*q* < 0.30
**50 µg/kg vs. Ctrl**	10	66
**100 µg/kg vs. Ctrl**	93	459
**Linear regression**	25	368

**Table 2:** Gene Ontology Biological Processes showing decreased phosphorylation on exposure with 50 µg/kg and 100 µg/kg MC-LR in Lepr^db^/J mouse liver samples relative to vehicle (PIANO—Platform for Integrative Analysis of Omics data, FDR < 0.1). Processes with decreased phosphorylation site abundance in microcystin exposed Lepr^db^/J liver samples relative to control (PIANO—Platform for Integrative Analysis of Omics data, FDR, False Discovery Rate < 0.1). A subset of the affected processes is shown here selected to reduce redundancy Results were summarized from *n* = 7–8 mice/group; 50 µg/kg MC-LR group (*n* = 8), 100 µg/kg MC-LR group (*n* = 7), vehicle (*n* = 8), for all proteomics analyses.

Pathway	Similar Pathways *^a^*	Mean *t*-Statistic	Sites	FDR
**Vehicle vs. 50 μg/kg MC-LR**				
Urogenital system development	18	−0.493	71	0.029
Regulation of T cell proliferation	9	−0.730	35	0.029
Appendage morphogenesis	9	−0.813	30	0.029
Regulation of DNA-binding transcription factor activity	7	−0.368	102	0.036
Regulation of striated muscle tissue development	7	−0.590	36	0.058
Regulation of cellular response to oxidative stress	6	−0.677	30	0.046
Energy derivation by oxidation of organic compounds	6	−0.460	92	0.029
Positive regulation of cell cycle process	6	−0.464	86	0.029
Defense response to other organism	5	−0.431	85	0.029
Response to wounding	4	−0.367	95	0.056
Cognition	4	−0.414	76	0.056
Regulation of microtubule cytoskeleton organization	4	−0.345	102	0.046
Monosaccharide metabolic process	3	−0.479	66	0.045
Protein autophosphorylation	3	−0.346	95	0.068
Lung alveolus development	3	−0.736	24	0.070
Coronary vasculature development	4	−1.032	17	0.029
**Vehicle vs. 100 μg/kg MC-LR**				
Toll-like receptor TLR6:TLR2 cascade	17	−1.073	32	0.0292
Transport of mature mRNA derived from an intron-less transcript	6	−1.257	21	0.0292
Cell cycle, mitotic	5	−0.555	132	0.0292
Resolution of sister chromatid cohesion	5	−1.111	30	0.0292
Mitotic prometaphase	4	−0.797	49	0.0426
Cytokine signaling in immune system	3	−0.580	144	0.0200
Carbohydrate metabolism	3	−0.717	105	0.0200
L1CAM interactions	2	−0.846	55	0.0292

*^a^* To reduce redundancy categories were clustered and one pathway per cluster listed. This indicates the size of the cluster.

**Table 3:** Identification of the clusters of pathways affected in 50 and 100 µg/kg MC-LR versus vehicle exposed Lepr^db^/J mice using Reactome database: Reactome pathways in liver were identified as affected by 50 as well as 100 µg/kg MC-LR versus db/vehicle (False Discovery Rate, FDR<0.2) and enriched in a c-means cluster (Fisher’s exact test *p* < 0.02). Results were summarized from *n* = 7–8 mice/group; 50 µg/kg MC-LR group (*n* = 8), 100 µg/kg MC-LR group (*n* = 7), Vehicle (*n* = 8), for all proteomics analyses.

Process	Mean *t*-Statistic	Sites	FDR
**Vehicle vs. 50 μg/kg MC-LR**			
Cluster 3			
Renal system development	−0.506	68	0.029
Regulation of stem cell proliferation	−0.838	31	0.029
Regulation of epithelial cell proliferation	−0.414	111	0.029
Regulation of mononuclear cell proliferation	−0.689	37	0.029
Regulation of leukocyte proliferation	−0.689	37	0.029
Regulation of T cell proliferation	−0.730	35	0.029
Positive regulation of cell cycle process	−0.464	86	0.029
Regulation of lymphocyte proliferation	−0.689	37	0.029
Urogenital system development	−0.493	71	0.029
Kidney development	−0.566	62	0.029
Coronary vasculature development	−1.032	17	0.029
Cluster 4			
Defense response to another organism	−0.431	85	0.029
Cluster 6			
Generation of precursor metabolites and energy	−0.375	133	0.029
**Vehicle vs. 100 μg/kg MC-LR**			
Cluster 1			
Pre-mRNA splicing	0.246	223	0.0601
mRNA splicing	0.246	223	0.0601
Cluster 2			
Post-translational protein modification	−0.31193	326	0.080192
Axon guidance	−0.30646	203	0.18749
Cluster 3			
Recruitment of mitotic centrosome proteins and complexes	−0.78878	24	0.14638
Cell-cell junction organization	−0.56824	45	0.15862
Centrosome maturation	−0.78878	24	0.14638
Toll-like receptor 4 (TLR4) cascade	−0.86804	41	0.042602
Signaling by interleukins	−0.57231	103	0.033413
Innate immune system	−0.37959	287	0.044106
Cytokine signaling in immune system	−0.58043	144	0.020048
Cluster 4			
Cellular senescence	−0.65136	49	0.082148

## Supplementary Materials

The following are available online at https://www.mdpi.com/2072-6651/11/9/486/s1, Figure S1: Effect of MC-LR on survival and gross liver morphology, Figure S2: Effect of MC-LR exposure on liver injury enzymes in NAFLD mice, Figure S3: Phosphorylation sites affected by MC-LR exposure, Figure S4: Fuzzy c-means clusters of phosphorylation site abundance versus microcystin dose. 6 clusters were generated, Table S1: Effect of MC-LR exposure on tissue weights in Lepr^db^/J mice, Table S2: Effect of MC-LR exposure on tissue weights in C57Bl/6J mice, Table S3: Effect of MC-LR exposure on blood chemistry in Lepr^db^/J mice, Table S4: Effect of MC-LR exposure on blood chemistry in normal C57Bl/6J (WT) mice, Table S5: Hematoxylin & Eosin (H&E), Periodic Acid-Schiff (PAS), and Composite Liver Injury Scores in Lepr^db^/J (db) mice, Table S6: Genetic analysis of hepatotoxicity in liver tissues, Table S7: Genetic analysis of oxidative stress response in liver tissues, Table S8: Identification of the clusters of pathways affected by 50 µg/kg MC-LR versus control using Reactome database, Table S9: GO Biological Process enrichment analysis, Table S10: REACTOME enrichment analysis, Table S11: Phosphorylation sites that were affected by microcystin exposure at 50 or 100 μg/kg.
