# Supplementary material for: Chronic Low Dose Oral Exposure to Microcystin-LR Exacerbates Hepatic Injury in a Murine Model of Non-Alcoholic Fatty Liver Disease
Source: Toxins (Basel). 2019 Aug 23;11(9):486. doi: 10.3390/toxins11090486 (PMC6783870; doi:10.3390/toxins11090486)
Supplement: Supplementary file 1 [file toxins-11-00486-s001.pdf]

Supplementary materials: Chronic Low Dose Oral Exposure to Microcystin-LR Exacerbates Hepatic Injury in a Murine Model of Non-Alcoholic Fatty Liver Disease

Apurva Lad, Robin C. Su, Joshua D. Breidenbach, Paul M. Stemmer, Nicholas J. Carruthers, Nayeli K. Sanchez, Fatimah K. Khalaf MBChB, Shungang Zhang, Andrew L. Kleinhenz, Prabhatchandra Dube, Chrysan J. Mohammed, Judy A. Westrick, Erin L. Crawford, Dilrukshika Palagama, David Baliu-Rodriguez, Dragan Isailovic, Bruce Levison, Nikolai Modyanov, Amira F. Gohara, Deepak Malhotra, Steven T. Haller and David J. Kennedy

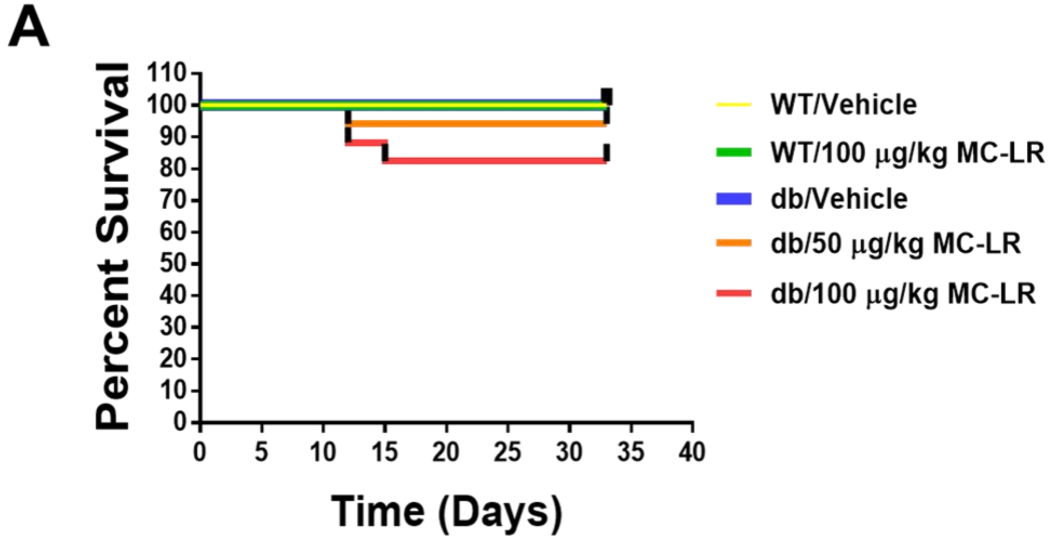

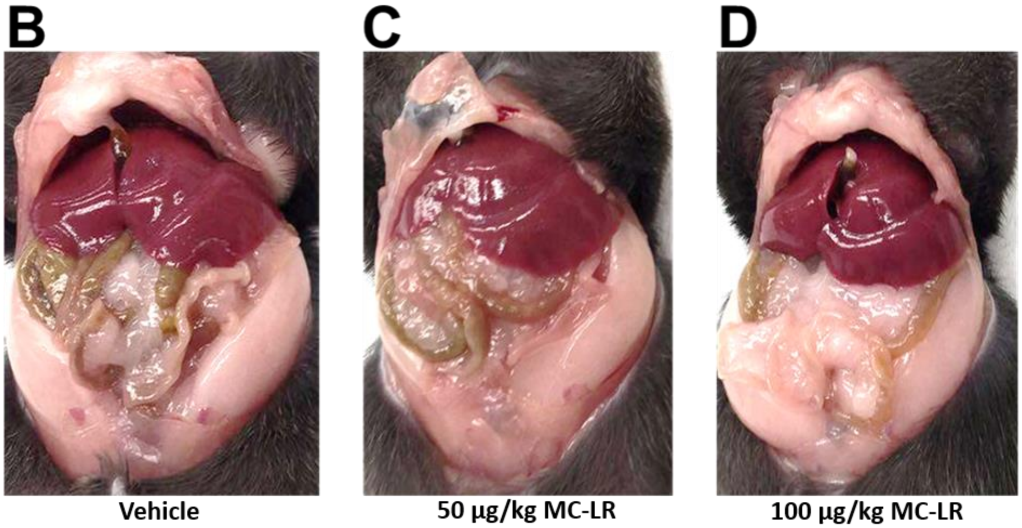

Figure S1. Effect of MC-LR on survival and gross liver morphology in both healthy (C57Bl/6J) and NAFLD (Lepr^{db}/J) mice. (**A**) Kaplan-Meier analysis of the survival period of the C57Bl/6J (WT) and LepR^{db}/J (db)

mice showed a non-significant (log-rank $p = 0.0702$) decrease in survival in mice receiving 50 µg/kg (n = 17, 94% survival) and 100 µg/kg MC-LR (n = 17, 82% survival) versus db/Vehicle (n = 15) (100% survival), no deaths were observed in the WT/Vehicle (n = 5) or WT/100 µg/kg MC-LR exposure (n = 5) C57Bl/6J mice; Representative images showing the gross morphology of the livers of Leprdb/J mice that were exposed to (**B**) Vehicle; (**C**) 50 µg/kg MC-LR or (**D**) 100 µg/kg MC-LR. In each case the animals died overnight and there was no observed acute trauma (e.g. tracheal rupture resulting in immediate death) or other signs of improper gavage technique such as visible signs of discomfort or bloating in the time preceding death. Full necropsies to determine cause of death were unable to be completed due to partial cannibalization by littermates, hence we are not able to fully exclude mortality attributable to the gavage procedure in these animals.

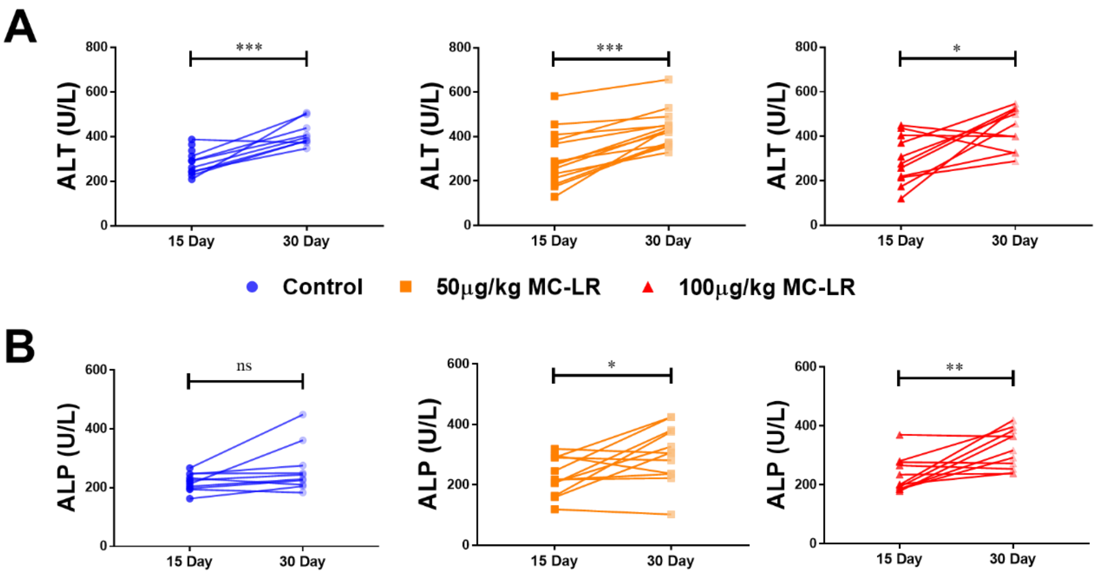

Figure S2. Effect of MC-LR exposure on liver injury enzymes in NAFLD (Lepr^{db}/J) mice. Circulating levels of (**A**) Alanine aminotransferase (ALT) were non-specifically elevated 30 days after study initiation across all exposure groups of LepR^{db}/J mice compared with 15 days after study initiation (db/Vehicle (n = 15) ***, $p < 0.001$, db/50 µg/kg MC-LR exposed (n = 16), ***, $p < 0.001$, db/100 µg/kg MC-LR exposed (n = 14), *, $p < 0.05$), while (**B**) Alkaline Phosphatase (ALP) were significantly elevated only in the MC-LR exposed groups as assessed at 15- and 30-days after study initiation (50 µg/kg MC-LR exposed *, $p < 0.05$, 100 µg/kg MC-LR exposed **, $p < 0.01$) via paired t-tests. "ns" denotes not significant.

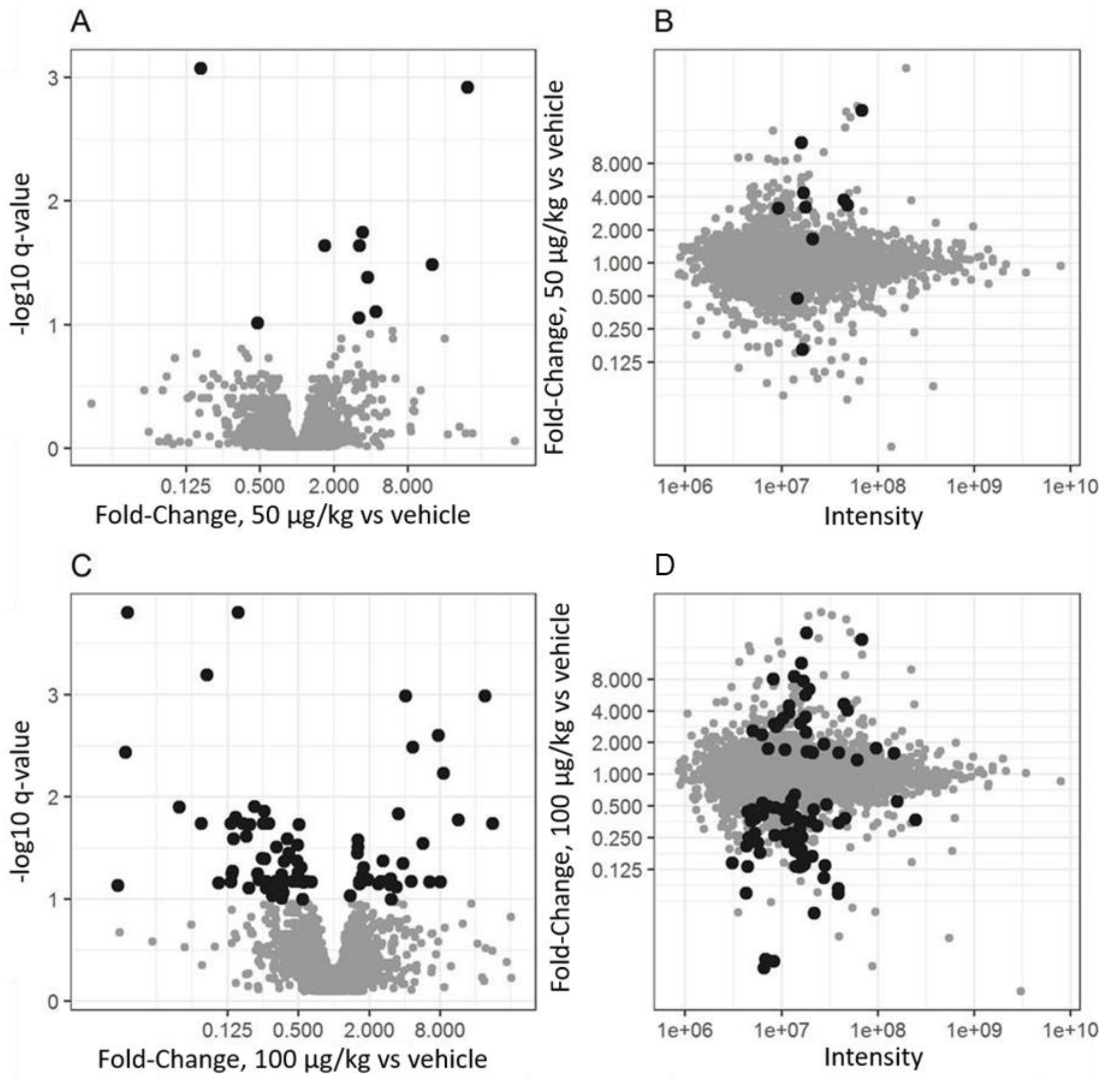

Figure S3. Phosphorylation sites affected by MC-LR exposure in NAFLD (Lepr^{db}/J) mice. Volcano plots (**A** and **C**) and minus-average plots (**B** and **D**). Values shown in parts A and B are comparisons of the 50 µg/kg microcystin exposed mice to vehicle controls and **C** and **D** are from the 100 µg/kg microcystin exposed vs vehicle controls. Sites that were affected by microcystin ($q < 0.1$) are highlighted in dark grey. 50 µg/kg MC-LR group ($n = 8$), 100 µg/kg MC-LR group ($n = 7$), Vehicle ($n = 8$), for all proteomics analyses.

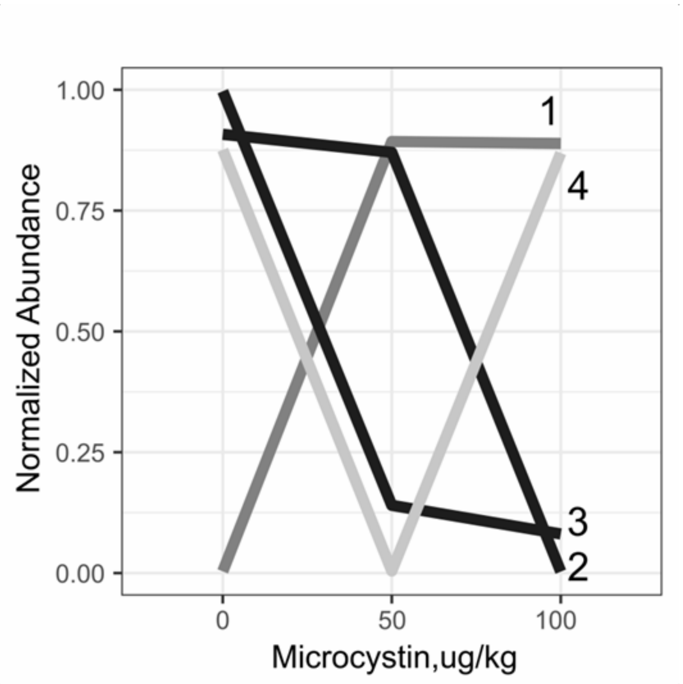

Figure S4: Fuzzy c-means clusters of phosphorylation site abundance versus microcystin dose in NAFLD (Lepr^{db}/J) mice. 6 clusters were generated. Only the 4 associated with Reactome pathways are shown, because the other two could not be interpreted biologically. Clusters that were dephosphorylated in microcystin exposed samples relative to unexposed are dark grey (clusters 2 and 3). Cluster 1 (medium gray) was hyperphosphorylated relative to unexposed samples. Cluster 4 (light grey) includes sites which were dephosphorylated at 50 µg/kg microcystin, but which were phosphorylated at 100 ug/kg microcystin. 50 µg/kg MC-LR group (*n* = 8), 100 µg/kg MC-LR group (*n* = 7), Vehicle (*n* = 8), for all proteomics analyses.

Table S1. Effect of MC-LR exposure on tissue weights in Lepr^{db}/J mice. The weights of the organs are measured in grams. One-way ANOVA was performed to determine the significance between the exposure groups.

Organ	db/Vehicle	db/50 µg/kg MCLR	db/100 µg/kg MCLR	
		Weight (grams) ± S.E.M.		ANOVA *p*-value
**Heart**	0.13 ± 0.02	0.148 ± 0.007	0.14 ± 0.004	0.74
**Lung**	0.11 ± 0.01	0.121 ± 0.005	0.121 ± 0.005	0.84
**Liver**	3.06 ± 0.22	3.242 ± 0.165	2.975 ± 0.148	0.58
**Kidney**	0.22 ± 0.04	0.202 ± 0.006	0.245 ± 0.026	0.47

Table S2. Effect of MC-LR exposure on tissue weights in C57Bl/6J mice. The weights of the organs are measured in grams. Unpaired t-test was performed to determine the significance between the exposure groups.

Organ	WT/Vehicle	WT/100 µg/kg MCLR	
	Weight (grams) ± S.E.M.		Unpaired *t*-test *p*-value
**Heart**	0.16 ± 0.01	0.16 ± 0.01	0.91
**Lung**	0.26 ± 0.01	0.27 ± 0.04	0.87
**Liver**	1.88 ± 0.13	1.42 ± 0.16	0.07
**Kidney**	0.19 ± 0.01	0.18 ± 0.01	0.91

Table S3. Effect of MC-LR exposure on blood chemistry in Lepr^{db}/J mice.

Group	Albumin (g/dL)	Alkaline phos-phatase (U/L)	Alanine amino-transferase (U/L)	Amylase (U/L)	BUN (mg/dL)	Non-fasted blood Glucose (mg/dL)	Total Protein (g/dL)	Globulin (g/dL)
**Day 15**								
Vehicle	4.5 ± 0.1	219 ± 8.4	287.6 ± 16.6	1294.2 ± 30.1	20.1 ± 0.9	426.4 ± 31	6 ± 0.1	1.5 ± 0.02
50 µg/kg MC-LR	4.3 ± 0.2	212.5 ± 21.7	302 ± 447.3	1251.4 ± 50.6	19.5 ± 0.7	400.2 ± 37.8	5.9 ± 0.2	1.5 ± 0.1
100 µg/kg MC-LR	4.3 ± 0.2	234.3 ± 17	293.8 ± 347.2	1212.2 ± 47.8	20 ± 0.9	450.8 ± 37.6	5.9 ± 0.2	1.5 ± 0.1
**Day 30**								
Vehicle	4.4 ± 0.2	263 ± 24.5	414.3 ± 17.7	1204.6 ± 59.4	20.9 ± 1.5	478.1 ± 44.0	6.1 ± 0.2	1.7 ± 0.1
50 µg/kg MC-LR	4.7 ± 0.2	301.8 ± 26	435.6 ± 304.8	1380.9 ± 68.7	20.4 ± 1.2	473.1 ± 32.8	6.5 ± 0.2	1.8 ± 0.1
100 µg/kg MC-LR	4.5 ± 0.1	322.4 ± 19.2	437.1 ± 296.7	1201.9 ± 75.5	23.4 ± 1.4	408.0± 40.0	5.4 ± 0.1	1.8 ± 0.1

Table S4. Effect of MC-LR exposure on blood chemistry in healthy C57Bl/6J (WT) mice.

Group	Albumin (g/dL)	Alkaline phos-phatase (U/L)	Alanine amino-transferase (U/L)	Amylase (U/L)	BUN (mg/dL)	Non-fasted blood Glucose (mg/dL)	Total Protein (g/dL)	Globulin (g/dL)
**Day 15**								
Vehicle	3.7 ± 0.1	70.8 ± 4.7	67.6 ± 17.8	780 ± 21.8	24.6 ± 0.8	275.8 ± 8.04	4.8 ± 0.1	1.1 ± 0.1
100 µg/kg MC-LR	3.1 ± 0.1	72.6 ± 4.9	52.4 ± 21.2	721.6 ± 20.2	20.6 ± 1.2	253 ± 15.3	5 ± 0.2	1.9 ± 0.1
**Day 30**:								
Vehicle	3.2 ± 0.1	58.4±8.0	59 ± 8.5	737.8 ± 47.6	24.4 ± 1.4	284.6 ± 13.8	4.8 ± 0.1	1.6 ± 0.1
100 µg/kg MC-LR	3.2 ± 0.1	58.2 ± 12.3	70.8 ± 17.5	730.0 ± 28.1	16.8 ± 0.9	204.6 ± 16.2	5.1 ± 0.2	1.9 ± 0.1

Table S5. Hematoxylin & Eosin (H&E), Periodic Acid-Schiff (PAS), and Composite Liver Injury Scores in Lepr^{db}/J (db) mice.

Exposure group	Mouse number	H&E Injury Score	PAS Injury Score	Composite Injury Score
**db/Vehicle**	1	2	1	1.5
	2	2	3	2.5
	3	2	2	2.0
	4	2	2	2.0
	5	2	2	2.0
	6	0	1	0.5
	7	2	3	2.5
	8	2	2	2.0
	9	2	3	2.5
	10	2	3	2.5
	11	2	3	2.5
	12	2	3	2.5
	13	2	2	2.0
**db/50 µg/kg MC-LR exposed**	1	4	4	4.0
	2	4	4	4.0
	3	4	4	4.0
	4	1	4	2.5
	5	2	1	1.5
	6	4	2	3.0
	7	4	4	4.0
	8	3	2	2.5
	9	3	2	2.5
	10	3	3	3.0
	11	2	3	2.5
	12	3	3	3.0
	13	3	4	3.5
	14	3	3	3.0
	15	*(Dead)*	*(Dead)*	*(Dead)*
	16	3	3	3.0
	17	2	2	2.0
**db/100 µg/kg MC-LR exposed**	1	2	3	2.5
	2	3	4	3.5
	3	2	4	3.0
	4	4	3	3.5
	5	2	3	2.5
	6	4	4	4.0
	7	*(Dead)*	*(Dead)*	*(Dead)*
	8	3	3	3.0
	9	2	2	2.0
	10	2	2	2.0
	11	*(Dead)*	*(Dead)*	*(Dead)*
	12	2	3	2.5
	13	*(Dead)*	*(Dead)*	*(Dead)*
	14	2	3	2.5
	15	3	2	2.5
	16	3	3	3.0
	17	3	3	3.0

Table S6. Genetic analysis of hepatotoxicity in liver tissues in NAFLD (Lepr^{db}/J) mice. Hepatotoxicity Array for gene expression of liver tissue homogenates from MC-LR exposed Lepr^{db}/J mice (db/100 µg/kg and db/50 µg/kg MC-LR exposure) relative to db/Vehicle exposed mice. qPCR arrays were run with liver cDNA from *n* = 4 pooled samples per array and *n* = 3 arrays per group. Student's t-test was used for comparison between MC-LR exposure group and vehicle control.

Gene	Description	50 µg/kg vs. Vehicle		100 µg/kg vs. Vehicle	
		Fold Regulation	*p*-value	Fold Regulation	*p*-value
	Cholestasis				
Abcc3	ATP-binding cassette, sub-family C (CFTR/MRP), member 3	174.2	0.091	123.3	0.214
Abcb1a	ATP-binding cassette, sub-family B (MDR/TAP), member 1A	26.6	0.132	15.8	0.365
Abcb4	ATP-binding cassette, sub-family B (MDR/TAP), member 4	95.1	0.123	46.1	0.258
Abcc2	ATP-binding cassette, sub-family C (CFTR/MRP), member 2	14.3	0.562	11.1	0.778
Atp8b1	ATPase, class I, type 8B, member 1	5.9	0.250	5.9	0.163
Pdyn	Prodynorphin	−480.5	0.368	−161.4	0.370
Rdx	Radixin	9.2	0.084	5.0	0.384
Slc51a	Organic solute transporter alpha	3.9	0.171	2.9	0.072
	Hepatotoxicity				
Aldoa	Aldolase A, fructose-bisphosphate	229.4	0.073	122.7	0.082
Apex1	Apurinic/apyrimidinic endonuclease 1	5.8	0.487	5.1	0.526
Avpr1a	Arginine vasopressin receptor 1A	5.5	0.322	7.4	0.090
Bhmt	Betaine-homocysteine methyltransferase	34.7	0.481	32.5	0.433
Btg2	B-cell translocation gene 2, anti-proliferative	3.4	0.509	3.1	0.511
Car3	Carbonic anhydrase 3	17.5	0.145	19.1	0.079
Casp3	Caspase 3	15.6	0.010	11.8	0.087
Ccng1	Cyclin G1	19.3	0.019	11.4	0.176
Cryl1	Crystallin, lambda 1	8.2	0.417	8.8	0.232
Cxcl12	Chemokine (C-X-C motif) ligand 12	170.3	0.008	150.0	0.085
Cyp1a2	Cytochrome P450, family 1, subfamily a, polypeptide 2	187.2	0.017	238.1	0.016
Ddit4l	DNA-damage-inducible transcript 4-like	19.7	0.115	46.3	0.364
Ddx39	DEAD (Asp-Glu-Ala-Asp) box polypeptide 39	33.3	0.098	54.7	0.230
Dnajb11	DnaJ (Hsp40) homolog, subfamily B, member 11	4.1	0.409	9.0	0.340
Dnajc3	DnaJ (Hsp40) homolog, subfamily C, member 3	1.5	0.358	2.1	0.232
Fads1	Fatty acid desaturase 1	2.3	0.129	2.4	0.135

Fxc1	Fractured callus expressed transcript 1	−1.1	0.989	−1.1	0.989
Gadd45a	Growth arrest and DNA-damage-inducible 45 alpha	5.1	0.054	9.0	0.366
Gclc	Glutamate-cysteine ligase, catalytic subunit	−2.1	0.386	−5.0	0.376
Gusb	Glucuronidase, beta	−1.3	0.390	−16.0	0.209
Hsp90ab1	Heat shock protein 90 alpha (cytosolic), class B member 1	−13.7	0.882	−17.4	0.835
Krt18	Keratin 18	27.3	0.094	6.4	0.356
Krt8	Keratin 8	18.8	0.258	14.8	0.423
Mbl2	Mannose-binding lectin (protein C) 2	18.9	0.574	19.2	0.568
MGDC	Mouse Genomic DNA Contamination	−12.5	0.374	−16.5	0.374
Nqo1	NAD(P)H dehydrogenase, quinone 1	9.3	0.136	4.0	0.387
Pla2g12a	Phospholipase A2, group XIIA	1.0	0.737	−2.8	0.118
Rb1	Retinoblastoma 1	2.5	0.044	2.0	0.108
Slc17a3	Solute carrier family 17 (sodium phosphate), member 3	2.5	0.963	2.4	0.881
Txnrd1	Thioredoxin reductase 1	3.5	0.707	3.5	0.707
	**Necrosis:**				
Cd68	CD68 antigen	5.0	0.460	6.4	0.193
Cdc14b	CDC14 cell division cycle 14 homolog B (S. cerevisiae)	−3.9	0.377	−3.2	0.379
Cdkn1a	Cyclin-dependent kinase inhibitor 1A (P21)	22.2	0.272	23.8	0.245
Col4a1	Collagen, type IV, alpha 1	33.2	0.025	36.1	0.229
Ddx39	DEAD (Asp-Glu-Ala-Asp) box polypeptide 39	33.3	0.098	33.3	0.098
Emc9	Family with sequence similarity 158, member A	1.5	0.311	1.5	0.311
Fam214a	CDNA sequence BC031353	15.6	0.013	19.5	0.011
Lgr5	Leucine rich repeat containing G protein coupled receptor 5	3.2	0.188	3.2	0.138
Mcm10	Minichromosome maintenance deficient 10 (S. cerevisiae)	14.1	0.188	3.3	0.371
Mlxipl	MLX interacting protein-like	97.1	0.016	46.3	0.207
Osmr	Oncostatin M receptor	3.5	0.416	3.5	0.416
Rhbg	Rhesus blood group-associated B glycoprotein	2.0	0.274	2.2	0.317
Serpine1	Serine (or cysteine) peptidase inhibitor, clade E, member 1	−2.4	0.933	−2.9	0.069
Skil	SKI-like	−1.3	0.649	−73.9	0.374
Tmem2	Transmembrane protein 2	−2.2	0.372	−2.2	0.372
	**Nongenotoxic Hepatocarcinogenicity:**				
Aldoa	Aldolase A, fructose-bisphosphate	229.4	0.073	122.7	0.082

Apex1	Apurinic/apyrimidinic endonuclease 1	5.8	0.487	5.1	0.526
Btg2	B-cell translocation gene 2, anti-proliferative	3.4	0.509	3.1	0.511
Ccng1	Cyclin G1	19.3	0.019	11.4	0.176
Cdkn1a	Cyclin-dependent kinase inhibitor 1A (P21)	22.2	0.272	23.8	0.245
Ddx39	DEAD (Asp-Glu-Ala-Asp) box polypeptide 39	33.3	0.098	54.7	0.230
Krt18	Keratin 18	27.3	0.094	6.4	0.356
Krt8	Keratin 8	18.8	0.258	14.8	0.423
Mrps18b	Mitochondrial ribosomal protein S18B	12.0	0.433	10.4	0.499
Txnrd1	Thioredoxin reductase 1	3.5	0.707	3.5	0.707
	**Phosholipidosis:**				
Abcb1a	ATP-binding cassette, sub-family B (MDR/TAP), member 1A	26.6	0.132	15.8	0.365
Asah1	N-acylsphingosine amidohydrolase 1	9.1	0.273	6.2	0.427
Fxc1	Fractured callus expressed transcript 1	−1.1	0.989	−1.1	0.989
Hpn	Hepsin	16.0	0.092	16.0	0.092
Lss	Lanosterol synthase	2.7	0.113	2.7	0.113
Mrps18b	Mitochondrial ribosomal protein S18B	12.0	0.433	10.4	0.499
S100a8	S100 calcium binding protein A8 (calgranulin A)	3.0	0.334	2.2	0.765
Serpina3n	Serine (or cysteine) peptidase inhibitor, clade A, member 3N	280.5	0.374	13.3	0.708
	**Steatosis:**				
Cd36	CD36 antigen	32.0	0.115	15.4	0.307
Fasn	Fatty acid synthase	38.6	0.156	22.9	0.328
Scd1	Stearoyl-Coenzyme A desaturase 1	−7.0	0.862	−2.7	0.740

Table S7: Genetic analysis of oxidative stress response in liver tissues of NAFLD (Lepr^{db}/J) mice. Oxidative Stress Array for gene expression of liver tissue homogenates from MC-LR exposed Lepr^{db}/J mice (db/100 μg/kg and db/50 μg/kg MC-LR exposure) relative to db/Vehicle exposed mice. qPCR arrays were run with liver cDNA from *n* = 4 pooled samples per array and *n* = 3 arrays per group. Student's t-test was used for comparison between MC-LR exposure group and vehicle control.

**Gene**	**Description**	**50 μg/kg vs. Vehicle**		**100 μg/kg vs. Vehicle**	
		Fold Regulation	*p*-value	Fold Regulation	*p*-value
	**Antioxidants:**				
	**Glutathione Peroxidases (GPx):**				
Gpx2	Glutathione peroxidase 2	3.4	0.378	1.1	0.826
Gpx5	Glutathione peroxidase 5	4.7	0.044	6.0	0.133
Gpx6	Glutathione peroxidase 6	4.0	0.855	3.7	0.753
	**Peroxiredoxins (TPx):**				
Ehd2	EH-domain containing 2	7.0	0.590	5.7	0.921
Prdx1	Peroxiredoxin 1	568.1	0.097	534.2	0.110
Prdx2	Peroxiredoxin 2	2876.3	< 0.001	3121.4	< 0.001
Prdx3	Peroxiredoxin 3	36.2	0.513	37.7	0.453
Prdx4	Peroxiredoxin 4	27.6	0.558	29.8	0.445
Prdx5	Peroxiredoxin 5	39.7	0.310	39.9	0.300
Prdx6	Peroxiredoxin 6	40.5	0.482	39.6	0.513
	**Other Peroxidases:**				
Apc	Adenomatosis polyposis coli	14.0	0.528	13.6	0.574
Cat	Catalase	68.4	0.616	77.1	0.441
Epx	Eosinophil peroxidase	−2.9	0.373	−5.9	0.235
Mpo	Myeloperoxidase	2.3	0.343	1.2	0.765
Ptgs2	Prostaglandin-endoperoxide synthase 2	1.9	0.455	−8.3	0.149
Serpinb1b	Serine (or cysteine) peptidase inhibitor, clade B, member 1b	4.3	0.236	1.1	0.815
Tpo	Thyroid peroxidase	−7.4	0.307	−6.8	0.309
	**Other Antioxidants:**				
Sod3	Superoxide dismutase 3, extracellular	2.1	0.435	2.5	0.267
	**Genes Involved in Reactive Oxygen Species (ROS) Metabolism:**				
	**Superoxide Dismutases (SOD):**				
Sod3	Superoxide dismutase 3, extracellular	2.1	0.435	2.5	0.267
	**Other Genes Involved in Superoxide Metabolism:**				
Cyba	Cytochrome b-245, alpha polypeptide	15.5	0.276	15.0	0.311
Ncf1	Neutrophil cytosolic factor 1	32.1	0.228	43.1	0.103
Ncf2	Neutrophil cytosolic factor 2	105.4	0.217	119.8	0.152

Nox4	NADPH oxidase 4	45.5	0.157	44.5	0.188
Noxa1	NADPH oxidase activator 1	32.5	0.327	34.0	0.293
Noxo1	NADPH oxidase organizer 1	14.2	0.580	32.1	0.093
Recql4	RecQ protein-like 4	4.8	0.355	1.5	0.629
Ucp2	Uncoupling protein 2 (mitochondrial, proton carrier)	−4.6	0.375	−4.7	0.375
**Oxidative Stress Responsive Genes:**					
Cat	Catalase	68.4	0.616	77.1	0.441
Epx	Eosinophil peroxidase	−2.9	0.373	−5.9	0.235
Gclc	Glutamate-cysteine ligase, catalytic subunit	54.4	0.164	40.4	0.416
Gpx2	Glutathione peroxidase 2	3.4	0.378	1.1	0.826
Gpx5	Glutathione peroxidase 5	4.7	0.044	6.0	0.133
Gpx6	Glutathione peroxidase 6	4.0	0.855	3.7	0.753
Hspa1a	Heat shock protein 1A	2.7	0.180	−1.2	0.499
Krt1	Keratin 1	13.3	0.218	1.0	0.898
Mpo	Myeloperoxidase	2.3	0.343	1.2	0.765
Nqo1	NAD(P)H dehydrogenase, quinone 1	236.7	0.071	197.8	0.125
Park7	Parkinson disease (autosomal recessive, early onset) 7	−1.2	0.598	28.4	0.965
Prdx1	Peroxiredoxin 1	568.1	0.097	534.2	0.110
Prdx2	Peroxiredoxin 2	2876.3	<0.001	3121.4	<0.001
Prdx6	Peroxiredoxin 6	40.5	0.482	39.6	0.513
Prnp	Prion protein	14.3	0.323	16.5	0.200
Psmb5	Proteasome (prosome, macropain) subunit, beta type 5	40.6	0.424	40.4	0.434
Tpo	Thyroid peroxidase	−7.4	0.307	−6.8	0.309
Txn1	Thioredoxin 1	3.7	0.296	4.1	0.199
Ucp3	Uncoupling protein 3 (mitochondrial, proton carrier)	6.6	0.375	9.7	0.342
**Oxygen Transporters:**					
Mb	Myoglobin	98.4	0.374	1.0	0.898
Ngb	Neuroglobin	11.4	0.074	10.6	0.030
**Other Reactive Oxygen Species (ROS) Metabolism Genes:**					
Il19	Interleukin 19	−16.4	0.141	−6.9	0.206

Table S8. Identification of the clusters of pathways affected by 50 µg/kg MC-LR versus control in NAFLD (Lepr^{db}/J) mice using Reactome database. Reactome pathways in liver were identified as affected by 50 µg/kg MC-LR versus control (False Discovery Rate, FDR < 0.2) and enriched in a c-means cluster (Fisher's exact test $p < 0.02$).

Pathway	Mean t-statistic	Sites	FDR
**Cluster 1**			
Processing of capped intron containing pre-mRNA	0.239	243	0.13031
Metabolism of RNA	0.191	403	0.13031
Pre-mRNA splicing	0.275	223	0.11026
mRNA splicing	0.275	223	0.11026
Sphingolipid metabolism	0.746	41	0.05012
Regulation of Actin dynamics for phagocytic cup formation	0.705	24	0.16038
**Cluster 2**			
ER to Golgi anterograde transport	0.539	83	0.020048
Asparagine N-linked glycosylation	0.442	98	0.064154
Transport to the Golgi and subsequent modification	0.520	84	0.026731
**Cluster 5**			
ER to Golgi anterograde transport	0.539	83	0.020048
Asparagine N-linked glycosylation	0.442	98	0.064154
Transport to the Golgi and subsequent modification	0.520	84	0.026731
Copii-mediated vesicle transport	1.149	36	0.020048

Table S9. GO Biological Process enrichment analysis in NAFLD (Lepr^{db}/J) mice.

Name	Test Statistic 50 μg/kg	FDR up, 50 μg/kg	FDR down, 50 μg/kg	Test Statistic, 100 μg/kg	FDR up, 100 μg/kg	FDR down, 100 μg/kg
Coronary vasculature development	−1.0323	1	0.039853	−0.75538	0.9996	0.35437
Embryonic limb morphogenesis	−0.9234	1	0.03282	−0.24075	0.9996	0.60095
Embryonic appendage morphogenesis	−0.9234	1	0.03282	−0.24075	0.9996	0.60095
Regulation of stem cell proliferation	−0.83803	1	0.03282	−0.36321	0.9996	0.48615
Regulation of glial cell differentiation	−0.83542	1	0.050215	−0.80835	0.9996	0.32969
Embryonic skeletal system development	−0.83197	1	0.050215	−0.57295	0.9996	0.4033
Appendage morphogenesis	−0.81328	1	0.03282	−0.1334	0.9996	0.71406
Limb morphogenesis	−0.81328	1	0.03282	−0.1334	0.9996	0.71406
Secondary metabolic process	−0.79835	1	0.090477	−0.74858	0.9996	0.35437
Regulation of reproductive process	−0.7809	1	0.060867	−0.33621	0.9996	0.54364
Kidney epithelium development	−0.75495	1	0.046495	−0.88391	0.9996	0.31733
Lung alveolus development	−0.73629	1	0.050215	−0.80052	0.9996	0.32969
Regulation of T cell proliferation	−0.72964	1	0.039853	−0.72412	0.9996	0.32969
Limb development	−0.70986	1	0.039056	−0.31014	0.9996	0.53082
Appendage development	−0.70986	1	0.039056	−0.31014	0.9996	0.53082
Regulation of mononuclear cell proliferation	−0.68949	1	0.03282	−0.72014	0.9996	0.31733
Regulation of leukocyte proliferation	−0.68949	1	0.03282	−0.72014	0.9996	0.31733
Regulation of lymphocyte proliferation	−0.68949	1	0.03282	−0.72014	0.9996	0.31733
Positive regulation of neuron apoptotic process	−0.68007	1	0.050215	−0.93462	0.9996	0.2897
Regulation of cellular response to oxidative stress	−0.67735	1	0.047824	−0.38762	0.9996	0.47396
Aerobic respiration	−0.67186	1	0.067418	−0.21038	0.9996	0.634
Tricarboxylic acid metabolic process	−0.66531	1	0.075278	−0.64745	0.9996	0.33792
Regulation of sister chromatid segregation	−0.65905	1	0.047824	−0.34456	0.9996	0.49524
Regulation of oxidative stress-induced cell death	−0.65798	1	0.078197	−0.45709	0.9996	0.45018
T cell differentiation in thymus	−0.65302	1	0.079111	−0.43452	0.9996	0.45298
Antibiotic metabolic process	−0.63816	1	0.03282	−0.61795	0.9996	0.32969
Cellular aldehyde metabolic process	−0.63584	1	0.074243	−0.46195	0.9996	0.42795
Cellular respiration	−0.63557	1	0.050215	−0.36331	0.9996	0.47936

Regeneration	−0.63153	1	0.097937	−0.34434	0.9996	0.52374
Regulation of response to oxidative stress	−0.62609	1	0.050215	−0.29387	0.9996	0.54364
Regulation of mitotic nuclear division	−0.60244	1	0.03282	−0.47837	0.9996	0.35519
Positive regulation of WNT signaling pathway	−0.59662	1	0.097937	−0.28342	0.9996	0.56835
Regulation of centrosome cycle	−0.5964	1	0.097937	−0.59589	0.9996	0.36526
Positive regulation of neuron death	−0.59283	1	0.039056	−0.66786	0.9996	0.32114
Regulation of striated muscle tissue development	−0.5898	1	0.050215	−0.54419	0.9996	0.35437
Regulation of muscle tissue development	−0.5898	1	0.050215	−0.54419	0.9996	0.35437
Regulation of muscle organ development	−0.5898	1	0.050215	−0.54419	0.9996	0.35437
Regulation of nuclear division	−0.5872	1	0.036707	−0.42514	0.9996	0.38427
Cell maturation	−0.58646	1	0.099436	−0.52822	0.9996	0.38824
Defense response to bacterium	−0.58604	1	0.097937	−1.1837	0.9996	0.2441
Regulation of chromosome segregation	−0.58367	1	0.041846	−0.36938	0.9996	0.4541
Lung morphogenesis	−0.57429	1	0.09821	−0.78393	0.9996	0.32969
Kidney development	−0.56637	1	0.03282	−0.51822	0.9996	0.32969
Regulation of lipid catabolic process	−0.56276	1	0.050215	−0.77661	0.9996	0.31733
Positive regulation of muscle tissue development	−0.54693	1	0.097937	−0.56794	0.9996	0.35654
Positive regulation of muscle organ development	−0.54693	1	0.097937	−0.56794	0.9996	0.35654
Positive regulation of striated muscle tissue development	−0.54693	1	0.097937	−0.56794	0.9996	0.35654
Response to interferon-gamma	−0.51951	1	0.056828	−0.26497	0.9996	0.56008
Regulation of DNA binding	−0.51891	1	0.099436	−0.34696	0.9996	0.49524
Renal system development	−0.50585	1	0.03282	−0.49304	0.9996	0.32969
Drug catabolic process	−0.50027	1	0.067418	−0.2297	0.9996	0.59712
Protein-DNA complex assembly	−0.49815	1	0.078197	−0.47807	0.9996	0.37118
Negative regulation of DNA-binding transcription factor activity	−0.49449	1	0.0547	−0.80439	0.9996	0.24798
Urogenital system development	−0.49283	1	0.041846	−0.48143	0.9996	0.32969
Memory	−0.49241	1	0.097937	−0.4507	0.9996	0.39629
Dicarboxylic acid metabolic process	−0.4887	1	0.080861	−0.10898	0.9996	0.73234
Positive regulation of mitotic cell cycle	−0.48853	1	0.046495	−0.32172	0.9996	0.4541
Cellular response to interferon-gamma	−0.488	1	0.097937	−0.29948	0.9996	0.53749
Monosaccharide metabolic process	−0.47864	1	0.039056	−0.40071	0.9996	0.37118
Response to hydrogen peroxide	−0.47562	1	0.063625	−0.41561	0.9996	0.39616
Regulation of organ growth	−0.4692	1	0.081699	−0.24578	0.9996	0.56323
Positive regulation of cell cycle process	−0.46384	1	0.03282	−0.34756	0.9996	0.38824

Energy derivation by oxidation of organic compounds	−0.46023	1	0.03282	−0.44166	0.9996	0.32969
Hindbrain development	−0.45555	1	0.078197	−0.47906	0.9996	0.35437
Response to light stimulus	−0.44092	1	0.039056	−0.32478	0.9996	0.43147
Learning	−0.4314	1	0.082513	−0.50844	0.9996	0.33792
Defense response to another organism	−0.43079	1	0.036707	−0.5297	0.9996	0.31733
Regulation of epithelial cell differentiation	−0.42359	1	0.074243	−0.085002	0.9996	0.77042
Regulation of epithelial cell proliferation	−0.41428	1	0.03282	−0.53129	0.9996	0.2897
Cognition	−0.4137	1	0.046495	−0.43928	0.9996	0.33792
Blood vessel morphogenesis	−0.4099	1	0.050215	−0.40647	0.9996	0.35437
Morphogenesis of a branching structure	−0.3965	1	0.09821	−0.3946	0.9996	0.39616
Positive regulation of developmental growth	−0.39581	1	0.056331	−0.36499	0.9996	0.38824
Embryonic organ morphogenesis	−0.39286	1	0.049009	−0.21415	0.9996	0.56404
Response to reactive oxygen species	−0.38934	1	0.085241	−0.36628	0.9996	0.41163
Hexose metabolic process	−0.38425	1	0.091131	−0.38871	0.9996	0.38824
Generation of precursor metabolites and energy	−0.37515	1	0.03282	−0.40782	0.9996	0.32969
Alpha-amino acid metabolic process	−0.37459	1	0.050215	0.14898	0.9996	0.97806
Regulation of DNA−binding transcription factor activity	−0.36756	1	0.039056	−0.58771	0.9996	0.2441
Response to wounding	−0.3666	1	0.049009	−0.34247	0.9996	0.38427
Regulation of developmental growth	−0.36623	1	0.039853	−0.32618	0.9996	0.38427
Innate immune response	−0.3635	1	0.03282	−0.35999	0.9996	0.33792
Cellular amino acid metabolic process	−0.35803	1	0.063625	0.11727	0.9996	0.97096
Heart morphogenesis	−0.35388	1	0.097937	−0.38264	0.9996	0.36832
Learning or memory	−0.35308	1	0.09821	−0.47487	0.9996	0.33477
Protein autophosphorylation	−0.34599	1	0.056828	−0.52372	0.9996	0.31733
Regulation of microtubule cytoskeleton organization	−0.34505	1	0.050215	−0.28394	0.9996	0.44346
Wound healing	−0.34439	1	0.089806	−0.35837	0.9996	0.388
Ribose phosphate biosynthetic process	−0.32493	1	0.097937	−0.40958	0.9996	0.33792
Activation of protein kinase activity	−0.32491	1	0.097821	−0.58535	0.9996	0.31733
Lung development	−0.32251	1	0.097937	−0.4715	0.9996	0.32969
Respiratory tube development	−0.32251	1	0.097937	−0.4715	0.9996	0.32969
Stress−activated protein kinase signaling cascade	−0.31809	1	0.097937	−0.3516	0.9996	0.39629
Immune effector process	−0.30741	1	0.067338	−0.44791	0.9996	0.32969
Regulation of microtubule-based process	−0.30347	1	0.079999	−0.24658	0.9996	0.48779
Cell junction assembly	−0.30113	1	0.099436	−0.29692	0.9996	0.44346

Epithelial tube morphogenesis	−0.29883	1	0.097937	−0.31585	0.9996	0.39616
Multicellular organismal homeostasis	−0.27123	1	0.091767	−0.2067	0.9996	0.54364
Positive regulation of growth	−0.26326	1	0.097937	−0.24782	0.9996	0.47936
Embryonic organ development	−0.25395	1	0.097937	−0.14627	0.9996	0.64959
Sulfur compound metabolic process	−0.25007	1	0.09821	−0.21717	0.9996	0.53134
Membrane docking	1.7117	0.13949	1	1.1412	0.34872	0.9997

Table S10. REACTOME enrichment analysis in NAFLD ($Lepr^{db}$/J) mice.

Name	Test Statistic, 50 µg/kg	FDR up, 50 µg/kg	FDR down, 50 µg/kg	Test Statistic, 100 µg/kg	FDR up, 100 µg/kg	FDR down, 100 µg/kg
Transport of mature mRNA derived from an intron less transcript	0.0058767	0.80688	0.95806	−1.2463	0.9998	0.034343
Transport of mature mRNAs derived from intron less transcripts	0.0058767	0.80688	0.95806	−1.2463	0.9998	0.034343
Amplification of signal from unattached kinetochores via a MAD2 inhibitory signal	−0.1484	0.89593	0.95806	−1.1858	0.9998	0.034343
Amplification of signal from the kinetochores	−0.1484	0.89593	0.95806	−1.1858	0.9998	0.034343
Mitotic spindle checkpoint	−0.1484	0.89593	0.95806	−1.1858	0.9998	0.034343
tRNA processing in the nucleus	0.26557	0.66085	0.95806	−1.151	0.9998	0.049062
Resolution of sister chromatid cohesion	−0.052978	0.85276	0.95806	−1.1431	0.9998	0.034343
Interactions of REV with host cellular proteins	−0.10241	0.85567	0.95806	−1.1382	0.9998	0.052566
Toll-like receptor TLR6:TLR2 cascade	−0.12584	0.89593	0.95806	−1.0464	0.9998	0.034343
Myd88:Mal cascade initiated on plasma membrane	−0.12584	0.89593	0.95806	−1.0464	0.9998	0.034343
Toll like receptor 2 (TLR2) cascade	−0.12584	0.89593	0.95806	−1.0464	0.9998	0.034343
Toll like receptor TLR1:TLR2 cascade	−0.12584	0.89593	0.95806	−1.0464	0.9998	0.034343
Rho GTPases activate formins	−0.14974	0.90799	0.95806	−1.0337	0.9998	0.038961
SNRNP assembly	0.014016	0.80688	0.95806	−1.0256	0.9998	0.052566
Non-coding RNA metabolism	0.014016	0.80688	0.95806	−1.0256	0.9998	0.052566
tRNA processing	0.15539	0.73985	0.95806	−0.99186	0.9998	0.056728
Sumoylation of chromatin organization proteins	0.049597	0.79947	0.95806	−0.98923	0.9998	0.079112
NS1 mediated effects on host pathways	0.035866	0.80688	0.95806	−0.98845	0.9998	0.079112
Interactions of VPR with host cellular proteins	−0.081569	0.85567	0.95806	−0.9872	0.9998	0.064672
Host interactions with influenza factors	−0.04111	0.83047	0.95806	−0.93593	0.9998	0.079875
Myd88 cascade initiated on plasma membrane	−0.082142	0.85567	0.95806	−0.86987	0.9998	0.064672
Toll like receptor 7,8 (TLR7, 8) cascade	−0.082142	0.85567	0.95806	−0.86987	0.9998	0.064672
Toll like receptor 9 (TLR9) cascade	−0.082142	0.85567	0.95806	−0.86987	0.9998	0.064672
Toll like receptor 10 (TLR10) cascade	−0.082142	0.85567	0.95806	−0.86987	0.9998	0.064672
Toll like receptor 5 (TLR5) cascade	−0.082142	0.85567	0.95806	−0.86987	0.9998	0.064672
Myd88 dependent cascade initiated on endosome	−0.082142	0.85567	0.95806	−0.86987	0.9998	0.064672
Traf6 mediated induction of NFκB and MAPKinases upon TLR7, 8 or 9 activation	−0.082142	0.85567	0.95806	−0.86987	0.9998	0.064672
Toll like receptor 4 (TLR4) cascade	−0.17957	0.94834	0.95806	−0.86876	0.9998	0.034343

Sumoylation of DNA damage response and repair proteins	−0.019179	0.8163	0.95806	−0.85362	0.9998	0.079112
Glycolysis	−0.20596	0.95309	0.95806	−0.83329	0.9998	0.034343
Neurotransmitter receptors and postsynaptic signal transmission	0.0099267	0.80688	0.95806	−0.82623	0.9998	0.079112
L1CAM interactions	−0.19482	0.95416	0.95806	−0.82517	0.9998	0.034343
Mitotic prometaphase	−0.097994	0.89593	0.95806	−0.82129	0.9998	0.034343
Toll-like receptors cascades	−0.17753	0.94834	0.95806	−0.82067	0.9998	0.056728
MAPKinase activation	−0.034018	0.83047	0.95806	−0.77462	0.9998	0.094619
Interleukin-17 signaling	−0.034018	0.83047	0.95806	−0.77462	0.9998	0.094619
Glucose metabolism	−0.30347	0.98499	0.95806	−0.77425	0.9998	0.038961
Gene silencing by RNA	0.25973	0.65381	0.95806	−0.77197	0.9998	0.094619
G alpha (q) signaling events	−0.011793	0.8163	0.95806	−0.75782	0.9998	0.079112
Transmission across chemical synapses	0.022646	0.80688	0.95806	−0.73544	0.9998	0.094619
Separation of sister chromatids	0.096406	0.77449	0.95806	−0.73402	0.9998	0.060346
Carbohydrate metabolism	−0.3009	0.9976	0.73593	−0.72332	0.9998	0.034343
Interleukin-1 family signaling	−0.16524	0.94834	0.95806	−0.71753	0.9998	0.079112
Toll like receptor 3 (TLR3) cascade	−0.068011	0.85567	0.95806	−0.71637	0.9998	0.094619
Trif(ticam1)-mediated tlr4 signaling	−0.061923	0.85567	0.95806	−0.70443	0.9998	0.089482
Myd88-independent tlr4 cascade	−0.061923	0.85567	0.95806	−0.70443	0.9998	0.089482
Cellular senescence	−0.20013	0.95309	0.95806	−0.66012	0.9998	0.079112
Host interactions of HIV factors	0.036324	0.80155	0.95806	−0.60512	0.9998	0.096406
Neuronal system	−0.040978	0.85567	0.95806	−0.56144	0.9998	0.082354
M phase	0.12954	0.66085	0.95806	−0.52435	0.9998	0.056728
Asparagine n-linked glycosylation	0.4424	0.036796	1	−0.29577	0.9998	0.42546
Transport to the Golgi and subsequent modification	0.52014	0.044156	1	−0.18367	0.9998	0.79725
ER to Golgi anterograde transport	0.53861	0.036796	1	−0.18155	0.9998	0.80716
Copii-mediated vesicle transport	1.1485	0.018398	1	0.23287	0.9998	0.95442
Sphingolipid metabolism	0.74576	0.018398	1	0.55462	0.80952	0.9979

Table S11. Phosphorylation sites that were affected by microcystin exposure at 50 or 100 μg/kg in NAFLD (Lepr^{db}/J) mice.

UniProt Accession	Protein Description	Phospho–site position	Phospho-site Amino Acid	Localiza-tion Class	*t*-statistic, 50 μg/kg vs control	*q*-value 50 μg/kg vs control	*t*-statistic, 100 μg/kg vs control	*q*-value 100 μg/kg vs control
P0C7T6	Ataxin-1-like	328	T	ClassI	−9.167754324	0.000815569	−9.753024961	0.000150532
Q8BTI8	Serine/arginine repetitive matrix protein 2	928	S	ClassI	5.586003855	0.02596753	6.500707487	0.001607155
Q8CIT0	Corticoliberin	73	S	ClassI	6.038618108	0.027730577	5.649087123	0.016548359
P26149	3 beta-hydroxysteroid dehydrogenase/Delta 5->4-isomerase type 2;3-beta-hydroxy-Delta (5)-steroid dehydrogenase; Steroid Delta-isomerase	53	S	ClassI	5.515083534	0.027730577	4.511294451	0.035022941
Q9Z0U1	Tight junction protein ZO-2	145	S	ClassI	7.017959912	0.037975325	6.58785868	0.019903452
Q8BTI8	Serine/arginine repetitive matrix protein 2	926	S	ClassI	4.895673186	0.046850529	5.858124769	0.004386913
Q8BSE0	Regulator of microtubule dynamics protein 2	53	T	ClassII	5.009656453	0.087089651	6.89945043	0.003607616
P57748	Matrix metalloproteinase-20	64	S	ClassI	4.892490726	0.096329393	4.093640888	0.076462084
Q9CQX2	Cytochrome b5 type B	37	T	ClassI	9.036986063	0.153355051	6.763922306	0.082338031
Q8CIT0	Corticoliberin	86	T	ClassI	4.383353013	0.157871237	3.969038721	0.081052099
Q6P4S6	Serine/threonine-protein kinase SIK3	493	S	ClassI	−3.931888098	0.204431476	−4.674863414	0.024351089
Q8C4X2	Casein kinase I isoform gamma-3	271	T	ClassI	−5.166797928	0.217599691	−5.489980669	0.066911022

P35831	Tyrosine-protein phosphatase non-receptor type 12	550	S	ClassI	−3.550367853	0.320967075	−4.388593243	0.040252248
P70441	Na(+)/H(+) exchange regulatory cofactor NHE-RF1	286	S	ClassII	4.287775135	0.356434364	9.675452547	0.007280685
Q9ERU9	E3 SUMO-protein ligase RanBP2	2729	S	ClassI	−3.552835511	0.356434364	−4.630854899	0.041150062
Q5XG73	Acyl-CoA-binding domain-containing protein 5	418	S	ClassI	−3.279739472	0.356434364	−4.068487104	0.053727985
P11983	T-complex protein 1 subunit alpha	544	S	ClassI	3.356286632	0.356434364	3.758522794	0.081052099
Q8CC35	Synaptopodin	672	S	ClassI	−3.092836063	0.395391087	−3.752820137	0.081052099
Q8VHR5	Transcriptional repressor p66-beta	135	S	ClassII	3.234946516	0.395391087	3.837008309	0.087038717
Q8BYC6	Serine/threonine-protein kinase TAO3	324	S	ClassI	−3.084193215	0.402018595	−4.053466047	0.068895259
Q8BTI8	Serine/arginine repetitive matrix protein 2	1832	S	ClassI	3.001399373	0.412525268	4.345598147	0.041150062
Q61081	Hsp90 co-chaperone Cdc37; Hsp90 co-chaperone Cdc37, N-terminally processed	13	S	ClassI	−2.825958934	0.467825565	−4.189869127	0.05163325
Q8BTI8	Serine/arginine repetitive matrix protein 2	2535	S	ClassI	2.714245165	0.478219815	4.193915254	0.046418872
Q8BTI8	Serine/arginine repetitive matrix protein 2	1225	S	ClassII	2.69441292	0.502605905	3.686022647	0.087038717
P51660	Peroxisomal multifunctional enzyme type 2;(3R)-hydroxyacyl-CoA dehydrogenase;Enoyl-CoA hydratase 2	3	S	ClassI	2.774174943	0.504562858	4.475592905	0.053196091
P28867	Protein kinase C delta type; Protein kinase C delta type regulatory subunit; Protein kinase C delta type catalytic subunit	645	S	ClassII	−3.227936825	0.504562858	−5.326986379	0.081052099

P18653	Ribosomal protein S6 kinase alpha-1	348	T	ClassII	−2.73992625 5	0.50590789 2	−4.632721 838	0.0515848 01
Q569Z6	Thyroid hormone receptor-associated protein 3	935	S	ClassI	−2.37785076 2	0.56971591 8	−3.837330 674	0.0810520 99
Q9QVP9	Protein-tyrosine kinase 2-beta	375	S	ClassI	−2.30491341 9	0.56971591 8	−3.721142 254	0.0810520 99
P24788	Cyclin-dependent kinase 11B	584	T	ClassI	−2.2959331	0.57932611 7	−3.723226 161	0.0810520 99
P98078	Disabled homolog 2	393	S	ClassII	2.46929904	0.60045070 5	−6.693720 307	0.0237185 06
Q9CZ44	NSFL1 cofactor p47	114	S	ClassI	−2.17228507	0.61030072 4	−3.638290 832	0.0837675 41
P24788	Cyclin-dependent kinase 11B	65	S	ClassI	−2.18456571	0.63247709 2	−4.413191 431	0.0464188 72
Q99L88	Beta-1-syntrophin	388	S	ClassI	−2.11730995 7	0.69009483 6	−5.006010 323	0.0349256 86
Q64735	Complement component receptor 1-like protein	454	S	ClassI	−1.92089503 6	0.72176907 9	−5.045062 234	0.0158141 82
Q5SZT7	NF-kappa-B-activating protein	141	S	ClassI	1.867301398	0.72423062 8	3.8103394 56	0.0810520 99
Q501J7	Phosphatase and actin regulator 4	264	S	ClassI	−1.73513327 9	0.76390831 9	−3.973485 431	0.0779384 74
Q3UQN2	F-BAR domain only protein 2	532	S	ClassI	−1.65311968 5	0.78657167 1	−6.120754 634	0.0219753 41
Q8K2L8	Trafficking protein particle complex subunit 12	343	S	ClassII	−1.45025654 8	0.79954299 5	−6.821162 211	0.0147738 48
A2ADY9	Protein DDI1 homolog 2	120	S	ClassII	−1.42473786 5	0.82453226 2	−6.668229 839	0.0340209 57
Q80U49	Centrosomal protein of 170 kDa protein B	881	S	ClassII	−1.34029148 4	0.82712687 2	−5.201899 116	0.0243510 89
Q62433	Protein NDRG1	330	S	ClassII	−1.29942601 6	0.86916035 1	−8.280938 473	0.0908769 72
O35623	BET1 homolog	50	S	ClassI	−1.03155168	0.90087827 8	−4.023962 258	0.0618286 4

Q8K0H1	Multidrug and toxin extrusion protein 1	18	S	ClassI	0.953298026	0.904348311	3.903857249	0.06997297
P26231	Catenin alpha-1	641	S	ClassI	0.857570987	0.917037344	4.016916843	0.06182864
Q5Y5T1	Probable palmitoyltransferase ZDHHC20	343	S	ClassII	−0.86634076	0.917339178	−11.89306365	0.000150532
P18653	Ribosomal protein S6 kinase alpha-1	373	T	ClassII	0.831109336	0.921224974	−5.714702657	0.017659411
O70472	Transmembrane protein 131	1153	S	ClassI	0.767365419	0.930964831	−4.865986751	0.024994405
Q69ZA1	Cyclin-dependent kinase 13	318	S	ClassI	0.755169748	0.931807531	−3.652618723	0.082338031
Q922J3	CAP-Gly domain-containing linker protein 1	199	S	ClassI	−0.678408116	0.937353445	−4.125208303	0.053196091
Q9QZW0	Phospholipid-transporting ATPase 11C	1107	S	ClassI	−0.674410101	0.937860875	−3.760551833	0.081052099
P70460	Vasodilator-stimulated phosphoprotein	235	S	ClassI	0.600316075	0.944834939	3.78783866	0.078717559
Q6ZQH8	Nucleoporin NUP188 homolog	1718	S	ClassII	0.574299295	0.948862166	−6.732854378	0.023718506
Q61469	Lipid phosphate phosphohydrolase 1	273	S	ClassII	0.561059382	0.948862166	−3.819070613	0.078717559
P48024	Eukaryotic translation initiation factor 1	17	S	ClassII	−0.513993806	0.957029854	−5.069176293	0.069738148
P20152	Vimentin	419	S	ClassI	−0.412885585	0.96527466	−3.853956429	0.081052099
Q58A65	C-Jun-amino-terminal kinase-interacting protein 4	203	S	ClassI	−0.427487994	0.96527466	−3.994386768	0.081052099
Q4VA53	Sister chromatid cohesion protein PDS5 homolog B	1359	T	ClassII	0.4563077	0.96527466	−6.803008444	0.082338031
Q8QZR5	Alanine aminotransferase 1	366	T	ClassII	−0.426903971	0.96527466	−4.715653121	0.093908917
P97360	Transcription factor ETV6	22	S	ClassI	-0.334452941	0.967590206	−3.920559433	0.081052099

Q80XU3	Nuclear ubiquitous casein and cyclin-dependent kinase substrate 1	79	S	ClassI	0.188935232	0.976097297	−4.147958263	0.06997297
Q63ZW7	InaD-like protein	455	S	ClassII	−0.116669259	0.977796017	−9.607922028	0.014773848
Q9Z0R4	Intersectin-1	315	S	ClassII	0.089161269	0.978676711	−15.20728177	0.004693039
Q9ERU9	E3 SUMO-protein ligase RanBP2	2087	S	ClassII	−0.074402056	0.978676711	−6.541475964	0.069738148
Q80T79	CUB and sushi domain-containing protein 3	1314	S	ClassII	0.002195801	0.982107982	−8.325525003	0.034925686
